# Biodiversity loss will decrease the future creditworthiness of nations

**DOI:** 10.1038/s41559-026-03081-7

**Published:** 2026-06-05

**Authors:** Matthew Agarwala, Matt Burke, Patrycja Klusak, Moritz Kraemer, Ulrich Volz, Benjamin K. Sovacool

**Affiliations:** 1 Bennett Institute for Innovation and Policy Acceleration, University of Sussex, Falmer, UK; 2 Department of Accounting and Finance, University of Sussex, Falmer, UK; 3 Bennett School of Public Policy, University of Cambridge, Cambridge, UK; 4 Tobin Center for Economic Policy, Yale University, New Haven, CT USA; 5 Sheffield University Management School, University of Sheffield, Sheffield, UK; 6 Edinburgh Business School, Heriot-Watt University, Edinburgh, UK; 7 Centre for Sustainable Finance, SOAS, University of London, London, UK; 8 Goethe-University, Frankfurt, Germany; 9 Department of Economics, SOAS, University of London, London, UK; 10 German Institute of Development and Sustainability (IDOS), Bonn, Germany; 11 Grantham Research Institute on Climate Change and the Environment, London School of Economics and Political Science, London, UK; 12 Centre for Economic Policy Research, London, UK; 13 Boston University, Boston, MA USA

**Keywords:** Economics, Development studies

## Abstract

Biodiversity loss and deforestation are increasingly recognized as systemic economic risks. Yet, their implications for financial markets remain poorly understood. Here we study how biodiversity and ecosystem service loss affect financial risk for the world’s largest asset class, sovereign debt. Environmental degradation undermines the natural foundations of economic activity, reducing productive capacity and the ability of governments to service debt. Currently, sovereign credit ratings ignore these risks, meaning that markets may be mispricing, mismanaging and misallocating US$83 trillion of financial assets. We incorporate biodiversity risk into sovereign credit assessments by extending S&P Global’s methodology to include scenarios for future tropical timber, wild pollination and marine fisheries services across 23 countries, representing 5.5 billion people. A partial ecosystem collapse scenario increases annual debt servicing costs by US$49 billion in India, equivalent to 2.4% of median post-tax income, and by US$70 billion in China. Across countries, additional annual interest payments could reach US$162 billion, nearly reaching the US$200 billion per year target for conservation support under the Global Biodiversity Framework. Angola, Bangladesh, the Democratic Republic of the Congo and Madagascar could face gross domestic product losses of more than 15% by 2030. Our results suggest that financial markets are systematically underpricing nature-related risks, with consequences for public finances, nature and financial stability.

## Abstract (web summary)

Scenario modelling reveals how sovereign credit ratings and the cost of borrowing across 23 nations would change under partial collapses in timber production, wild pollination services and marine fisheries.

## Main

With at least half of global gross domestic product (GDP) considered moderately or highly dependent on nature^1^, there is mounting evidence that environmental–economic risks extend well beyond the climate system to include biodiversity loss, deforestation, water stress and broader environmental change^2,3^. The near-term human, economic and financial toll of nature loss are substantial. Deforestation and species loss alone make pandemics such as COVID-19 more likely^4^, and estimates indicate that even partial collapses in just three ecosystem services—wild pollination, marine fisheries and tropical timber—could result in “a decline in global GDP of US$2 trillion annually by 2030”^2^.

Yet, action on biodiversity policy and finance has long lagged behind climate efforts. The Intergovernmental Panel on Climate Change was established in 1988, whereas the Intergovernmental Science-Policy Platform on Biodiversity and Ecosystem Services was established only in 2012. The Stern Review on the economics of climate change arrived 15 years before the Dasgupta Review on biodiversity, and the Task Force on Climate-related Financial Disclosures predates its nature-related counterpart (Taskforce on Nature-related Financial Disclosures) by 6 years. Meeting the Kunming–Montreal Global Biodiversity Framework (GBF)’s target to mobilize US$200 billion per year for biodiversity^5^ will require scientifically rigorous assessments of nature-related financial risks.

Macroeconomic studies on biodiversity often focus on global assessments of the value of natural capital and ecosystem services to human well-being^6–9^ rather than quantifying risks to financial assets associated with nature loss. By contrast, climate finance research has assessed aggregate global financial risks^10^, or identified global exposure to specific risks such as asset stranding^11^.

Biodiversity finance, however, is a much younger field^12^. Pioneering research describes the conditions under which public and private capital might support conservation^13^, the amount of finance needed to meet targets set out under the Kunming–Montreal GBF^14^, or the extent to which investor expectations about biodiversity risk affect current asset prices^15,16^.

Such focus from finance specialists on biodiversity-related risks is welcome, but one concern is how well the resulting research incorporates environmental science. Knowing the characteristics of existing biodiversity-finance deals^13^, or how investors react to news about biodiversity risk^15,16^, is critically important. However, such deals and reactions may have little relationship to underlying ecological fundamentals. Editors may choose newspaper headlines to drive clicks and sell adds. Contracts for biodiversity finance are not evidence of conservation outcomes. Neither is a substitute for science.

We incorporate environmental science into biodiversity finance by using ecological–economic models to identify and price biodiversity- and ecosystem-related financial risks for the world’s largest asset class: sovereign debt. Our approach embeds forward-looking scenarios of ecological shocks into existing assessments sovereign creditworthiness. We focus on sovereign debt because, at over US$83 trillion^17^, it is a primary source of public investment, its price (the interest rate paid by governments on public borrowing) is a key determinant of development potential^18^, and it is the ‘safe asset’ to which markets turn in times of turmoil (flight to safety). However, in many countries, interest on debt can become a severe constraint on public finances, with disastrous consequences for sustainable development^19^. While governments hope that private markets will mobilize substantial financial flows in pursuit of nature conservation, the public goods nature of ecosystems means that public finance needs to play a dominant role^20–22^.

For-profit credit ratings agencies (CRAs) produce sovereign credit ratings to provide markets with information about sovereign creditworthiness, assessing the ability and willingness of sovereigns to service debt. Such ratings affect the cost of borrowing faced by countries, and therefore the allocation of capital globally. When ratings overlook large, novel sources of risk—as in the subprime mortgage crisis in the USA, which led to the 2008 global financial crisis—the consequences can be globally catastrophic. Against mounting scientific and economic evidence, the omission of nature-related financial risks from sovereign ratings becomes increasingly problematic.

Among ‘the big three’ CRAs, none of the ratings methods explicitly incorporates financial risks arising from the future loss of biodiversity and ecosystem services^23–26^. However, all are capable of indirectly incorporating the consequences of past—backward-looking—risks. For instance, if historical biodiversity loss reduces GDP, or increases debt-to-GDP ratios, this would be factored into current ratings. However, a metric that only reveals risk ‘after the fact’ is of little use to financial markets, which must allocate capital today in pursuit of returns tomorrow. The difference between forward- and backward-looking risk measures is like the difference between a doctor’s diagnosis beforehand versus an autopsy report afterwards: they reveal an opportunity to act.

Although leading CRAs are actively engaged in discussions with scientists, economists, data providers, environmental non-governmental organizations and international financial institutions to consider how nature loss might affect creditworthiness, no methodological change has been introduced so far^23–26^.

To incorporate biodiversity loss within sovereign credit assessment, we first construct a model to predict current sovereign credit ratings. We then incorporate forward-looking projections of macroeconomic outcomes under a range of environmental–economic scenarios to simulate the effect of nature loss on ratings (Supplementary Fig. 1).

Building on the latest research into the determinants of sovereign ratings^27–29^, we focus on S&P’s long-term foreign-currency sovereign ratings because S&P is one of the largest CRAs, issues ratings covering the most sovereigns over the longest time period and is recognized as a market leader; moreover, their published methodology is among the most transparent and replicable^25,30^. Our non-parametric ratings prediction model incorporates six macroeconomic variables, including: GDP per capita, GDP growth, net general government debt/GDP, general government balance/GDP, narrow net external debt/current account receipts (CAR) and current account balance/GDP.

## Results

We present results in terms of three indicators that are familiar to markets and regulators: sovereign credit ratings, default probability and the cost of borrowing. The link between biodiversity loss and these indicators is a priori unknown. In the short run, converting natural capital into income (for example, by logging tropical forests) could appear to reduce credit risk by increasing GDP and exports, and by lowering debt-to-GDP and external debt-service-to-exports ratios. However, in the longer run, this reduces the natural capital stock, and therefore the productive capacity of the economy, ultimately increasing risk, reducing creditworthiness and making public borrowing (and investment) costlier because lenders demand higher risk premia^31^.

### A framework for integrating biodiversity loss with credit ratings

To simulate the effect of future nature loss on ratings, we develop an integration pipeline that maps GTAP-InVEST scenario outputs to country-specific macroeconomic pathways and embeds them into sovereign credit ratings, default probabilities and the cost of borrowing for 23 sovereigns (Methods). GTAP-InVEST is the first-of-its-kind effort to combine a global, spatially explicit ecosystem service valuation model (Integrated Valuation of Ecosystem Services and Trade-offs, InVEST) with a global computable general equilibrium model (Global Trade Analysis Project, GTAP) to jointly determine how changes in natural capital and ecosystem service provision affect primary industries, scale through value chains, and spread through trade links, to affect macroeconomic outcomes such as GDP.

Under a range of scenarios, GTAP-InVEST estimates GDP losses associated with declines in the provision of multiple ecosystem services: marine fisheries, wild pollination services and tropical timber production. The choice of these ecosystems services is predetermined by their ability to be modelled endogenously as sectoral productivity shocks that meet two conditions: (1) the availability of globally consistent, spatially explicit data; and (2) a clear functional link between biophysical change and sectoral economic productivity (Methods). Although other InVEST modules exist, such as water yield, flood regulation and soil retention, the lack of harmonized global data and the difficulty of consistently mapping them to market sectors prevent their integration into the coupled computable general equilibrium (CGE) model. We would expect the inclusion of additional components of biodiversity and ecosystem services to affect the magnitude and relative geographic distribution of our results, although these can be devilishly difficult to model.

At the time of writing, this three-service specification remains the field standard in global CGE-linked environmental–economic modelling^2^. Our results reflect two GTAP-InVEST scenarios. Specifically, we consider a business-as-usual (BAU) scenario that ignores ecosystem service decline against one that incorporates ecosystem service loss (BAU-ES). Under BAU-ES, habitat loss and economic activity continue to degrade natural capital and ecosystem services at their current rate, and global GDP in 2030 is US$75 billion (2014 US dollars) less than in the counterfactual BAU world (in which declining natural capital and ecosystem services are excluded from the model). We additionally explore a partial ecosystem collapse (PEC) scenario, which entails a 90% reduction in wild pollination services, a 90% reduction in total catch biomass from marine fisheries, and a conversion of 88% of tropical forest into grassland or shrubland. The result is a shortfall in global GDP of US$2 trillion (2014 US dollars) annually, compared with the BAU scenario^2^.

The approach means that our results reflect scenario analyses, not predictions of future ratings. Predictions would require probabilistic statements about the likelihood of each scenario, which are not presently available. The PEC scenario serves as a plausible tail-risk stress test, as is common in environmental economics^32^ and practitioner reports^33,34^. For context, a 90% collapse scenario is consistent with research on pollinators^35–37^ and observable history in marine fisheries^38^. There is greater debate over the magnitude and timing of collapse in tropical forests^39–43^.

Aggregating these economic losses to the country level allows forward-looking scientific evidence on the economic consequences of ecosystem service loss to be directly integrated into sovereign credit risk assessment.

### Impact of biodiversity loss on sovereign credit ratings

**Fig. 1:**
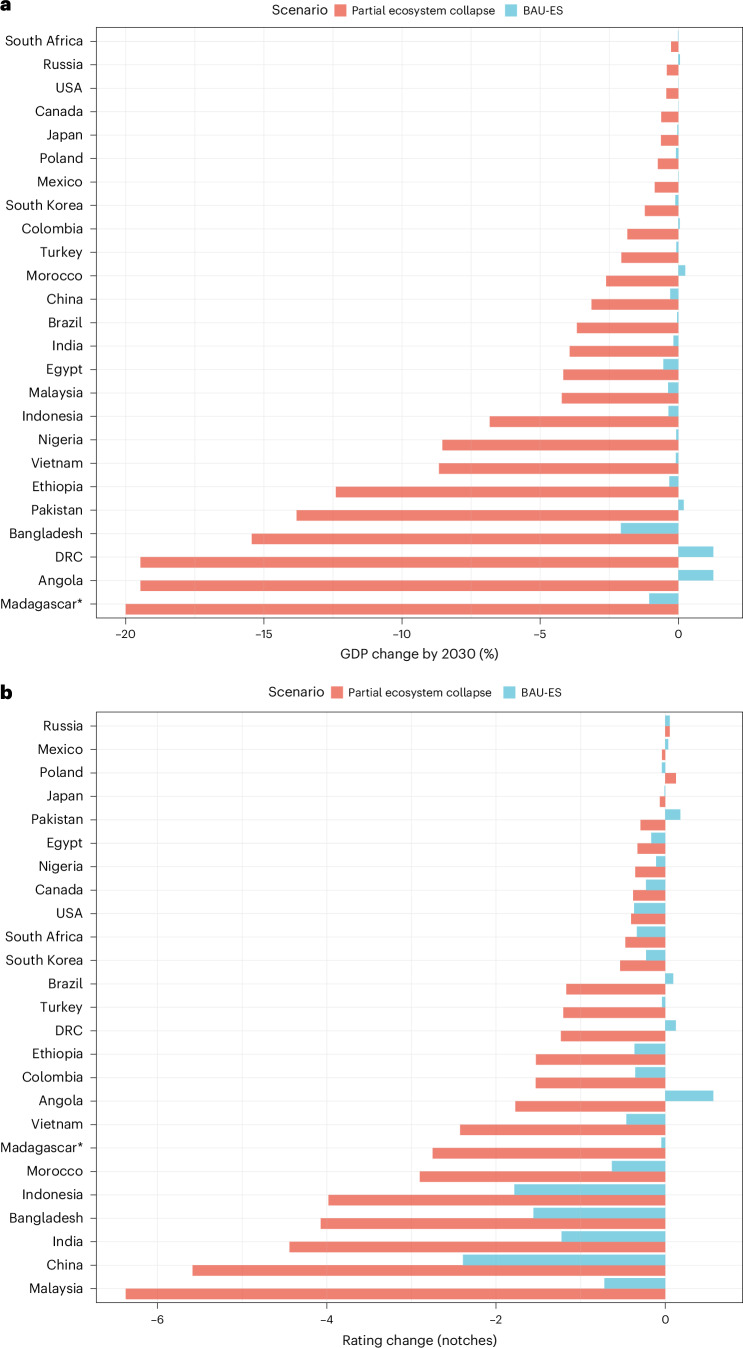
Credit rating changes under current trends and partial-collapse scenarios. **a**, Percentage shortfall in GDP under a scenario in which current rates of natural capital and ecosystem decline persist through 2030 (BAU-ES, blue) and under a PEC (red). **b**, Impact of BAU-ES and PEC scenarios on sovereign credit ratings. *Madagascar is a special case, as S&P Global did not issue a rating for this country during the 2015–2020 training sample period. To retain Madagascar within our sample, we used our credit ratings prediction model to estimate a synthetic rating for Madagascar, which we later adjust for biodiversity loss (‘Adjusting ratings factors for environmental risk’ section in Methods).

Figure 1a reports country-level percentage changes in GDP resulting from natural capital and ecosystem service loss under the BAU-ES and PEC scenarios, as estimated by GTAP-InVEST^2^. While most countries in the sample experience only minor economic damages under the BAU-ES scenario, Bangladesh experiences a shortfall of 2% of GDP. By contrast, Angola and the Democratic Republic of the Congo (DRC) see GDP gains of 1.3% as broader economic growth outpaces losses associated with ecosystem decline under the BAU-ES scenario in these countries.

By contrast, the PEC scenario reduces GDP across the sample. As expected, the impacts are larger for countries where output depends most heavily on tropical timber, marine fisheries and wild pollination services, but even countries such as Poland (which produces no tropical timber) are affected through trade relationships.

Figure 1b translates GDP losses under each scenario into impacts on sovereign credit ratings. Even under the BAU-ES scenario, downgrades of at least one notch on the 20-notch scale are expected in Indonesia, Bangladesh, India and China. The PEC scenario entails much more severe downgrades, with downgrades of at least four notches in Malaysia, China, India and Bangladesh. Under this scenario, the DRC, Angola and Madagascar become unratable, as their simulated ratings fall below the lowest grade found in the training data. In practical terms, this scenario would probably lead to a sovereign default.

The results in Fig. 1 reveal an important feature of the nature–GDP–credit ratings relationship, which is highly nonlinear. This is demonstrated in the rankings of countries by impacts on GDP versus credit ratings. Nonlinearity in the GDP–ratings relationship arises because ratings are constructed using discrete ‘income bands’. Countries near band thresholds can experience large rating changes from modest GDP shocks, whereas countries in the middle of a band show limited sensitivity. Our model recovers these threshold effects directly from the ratings data, explaining the observed cross-country heterogeneity. In terms of impacts on GDP, China and Malaysia are in the middle of the pack, with shortfalls of 3.1% and 4.2%, respectively (Fig. 1a), but these translate into the greatest impacts on sovereign downgrades, with China and Malaysia sliding 5.5 and 6.3 notches, respectively (Fig. 1b). Finally, because these results reflect losses across only a limited range of ecosystem services, the associated impacts should be considered lower bounds.

### Impact on default probability and borrowing costs

**Fig. 2:**
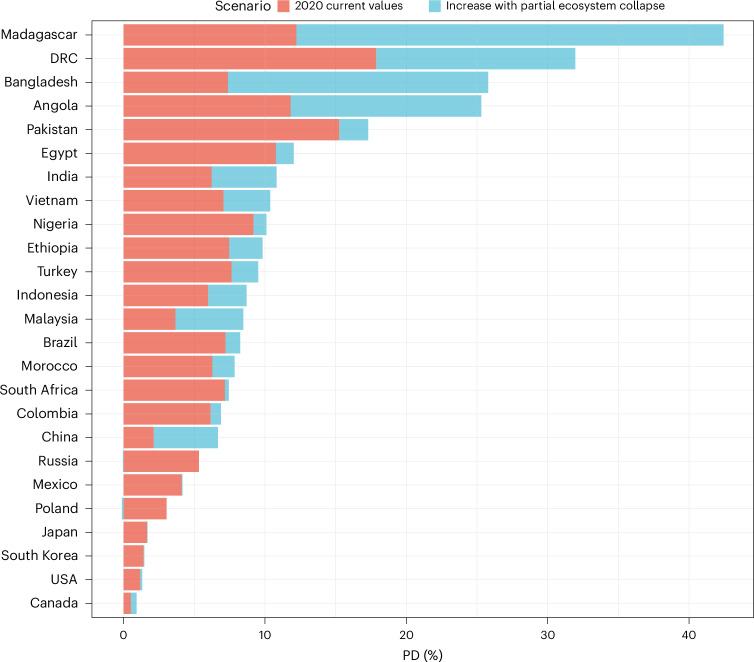
Nature-related increases in PD. PD by country, current (red), additional due to PEC (blue), and combined, following partial collapse (red + blue). Current PD values are derived from current sovereign ratings and the historical default probabilities associated with those ratings.

Credit ratings effectively convey an analytical assessment of a sovereign’s probability of defaulting on debt. These probabilities of default (PD) are reported in Fig. 2. Current PDs for each country, ignoring any future impacts from declining natural capital or ecosystem services, are reported in red. Madagascar, the DRC, Bangladesh, Angola and Pakistan are most at risk of PD. However, nature loss increases sovereign risk, with the associated increase in PD shown in blue. These are additive, meaning that China’s PD under the PEC scenario (6.7%) would be the sum of current PD (2.1%) and the additional, nature-driven PD (4.5%). Thus, this scenario entails a threefold increase in the probability of Chinese default.

**Fig. 3:**
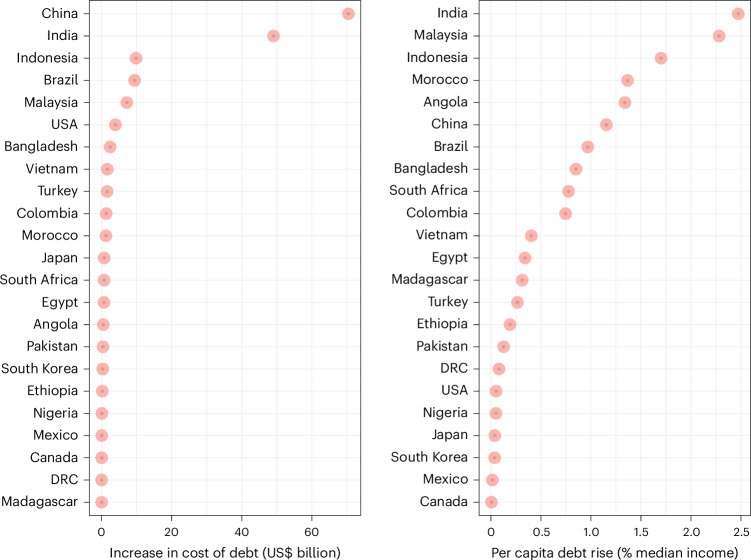
Additional annual debt servicing costs due to PEC. Effect of the PEC scenario on annual debt servicing costs. Left: increase in annual debt interest payments in billions of US dollars. Right: increase in annual debt interest payments as a percentage of median after-tax income.

Finally, we show the effect of a partial nature collapse on annual debt servicing payments. Figure 3 plots the additional annual interest payments on outstanding debt under the PEC scenario. China and India face the greatest economic exposure, with additional annual interest payments of US$70 billion and US$49 billion, respectively. Across just the 23 countries in the sample, we find an additional annual interest payment of US$162 billion. This is a substantial amount concentrated in a very small number of countries. For comparison, the combined net interest payment across all developing countries in 2022 was US$847 billion (ref. ^19^). Put differently, global development aid in 2023 was US$223.7 billion according to the Organisation for Economic Co-operation and Development, meaning that these interest payments are equivalent to 72.4% of all overseas development aid worldwide^44,45^.

### Policy options for meeting additional interest payments

There are four ways through which countries might meet these additional interest payments. First, countries could cut public spending elsewhere, reallocating resources towards debt servicing costs but away from mass transit infrastructure, education, healthcare or national security. However, in many developing countries, debt servicing already commands a dominant share of public expenditure. Globally, more than 3.3 billion people live in countries that spend more on interest than on education or health^19^.

A second option is to borrow more to meet current payments. However, this merely delays the problem as additional debt today must be serviced tomorrow. Moreover, the rates at which many biodiversity-rich developing countries borrow are already two to four times higher than in the USA^19^, and our results indicate that continued nature loss will only exacerbate the spread.

A third option is simply for sovereigns to default, but global debt markets are notoriously unpredictable and a history of sovereign default imposes substantial costs on future borrowing.

Fourth, the additional interest payments could be met by raising taxes. Figure 3 (right) offers an indication of what this might look like for the median citizen. Here, India’s US$49 billion of additional interest (Fig. 3, left) is converted into international dollars (to adjust for differences in the cost of living across countries) and divided by median after-tax income. Thus, the additional interest payment arising from nature loss would be equivalent to 2.4% of the median Indian’s disposable income, each year.

## Discussion

The continued destruction of natural capital and loss of associated ecosystem services could yield catastrophic financial risks worldwide. We use ecosystem service degradation as a partial and conservative transmission channel linking biodiversity loss to impacts on creditworthiness. While biodiversity encompasses genetic, species and ecosystem-level dimensions^46^, many of which may decline without immediate economic effects, our results suggest that declines in just these three (tropical timber, marine fisheries and wild pollination) services could be financially material. As natural capital continues to decline^9^, credit ratings that ignore scientific information about nature-related risks may obscure financially material vulnerabilities. We calculate the impacts of losses in marine fisheries catch, tropical timber and wild pollination services on sovereign credit ratings, the cost of public borrowing and the probability of sovereign default under a BAU and partial collapse scenario.

Although our study covers 23 countries, it includes India, China, the USA, Indonesia and Pakistan, with a combined population of about 5.5 billion people. Across the sample, the lower-bound estimate of a combined additional debt servicing costs would be US$162 billion—every year—just to cover the additional risk premium on public debt due to nature loss. For comparison, the Kunming–Montreal GBF signed by 196 countries set a target of mobilizing US$200 billion per year globally for conservation^5^. India and China are among the most exposed, facing a potential increase in annual interest payments of US$49 billion and US$70 billion, respectively.

For the credit ratings industry, our results demonstrate that the effects of nature loss on rating fundamentals can be identified in a scientifically rigorous manner, that they are large and financially material, and that they can be incorporated into sovereign credit risk assessment.

For investors, we show that forward-looking environmental risks can be incorporated into mainstream risk measures such as credit ratings, without the need to resort to poorly defined ‘ESG’ metrics that often lack scientific underpinnings.

We argue that governments, central banks, financial regulators and international financial institutions such as the World Bank and International Monetary Fund cannot afford to ignore nature-related financial risk. Given the likely impact of nature loss on output, sovereign ratings and debt, credit rating agencies ought to explicitly include these risks into their ratings methodologies. Conceptually, incorporating biodiversity- and nature-related risks into sovereign ratings is no different from incorporating other highly uncertain risks such as geopolitical risk. Indeed, the risk of biodiversity loss can be precisely quantified and geographically localized.

Finally, we show that the additional interest payments associated with increased sovereign risk due to even a PEC could be substantial. Indeed, the impacts across the 23 countries could lead to additional interest payments of US$162 billion per year, or 81% of the total annual target for biodiversity finance to be raised by all countries under the GBF. If countries are putting such scarce resources into paying interest, they are forgoing the opportunity to make critical investments into ecosystem services, water or nature-based protection. Ultimately, when it comes to the impact of biodiversity loss on sovereign debt, economies face a choice: pay now, by investing in nature, or pay later through reduced fiscal space, higher borrowing costs and higher interest payments that crowd out other growth-enhancing investments. The ‘pay now’ option generates long-term returns for people, business and nature. The ‘pay later’ option entails large downside risks, with little to no upside.

Although our modelling presents a range of estimations, the overall finding is stark: sovereign nations (and ultimately their markets and taxpayers) may face substantial financial costs due to nature loss. Debt in lower-income, often biodiversity-rich countries that already face among the world’s highest borrowing costs may be especially vulnerable to higher risk premia driven by nature loss^21^. The UN Secretary General describes the ‘crushing’ debt crisis as ‘nothing less than a development disaster’^47^. We show that continued nature loss will only exacerbate the challenge, undermining the ability of many biodiversity-rich nations to meet development goals across the board, from education, health and infrastructure to investments in nature conservation and climate adaptation. Moreover, higher borrowing costs are not restricted to governments: well-known dynamics known as the ‘sovereign ceiling’^30^ and ‘sovereign spillover’^48^ effects mean that higher borrowing costs for sovereigns lead to higher borrowing costs for banks and corporates in the affected countries.

### The future of biodiversity finance

We welcome the growing recognition of the need to invest in natural capital, ecosystem services and nature conservation^14^, but progress in mobilizing biodiversity finance has been slow^12,20^. Biodiversity finance—as a research field and in practice—faces several obstacles, including a proliferation of potentially confusing metrics^13,49^, the difficulty of capturing private financial returns to the provision of public environmental goods and services^13^, and difficulties in turning scientific information about the myriad economic values generated by nature^50^ into specific insights about material risks on which investors can act.

As the field of biodiversity finance evolves, it is critically important to ensure that environmental science is integrated from first principles. Our research indicates that it is possible to incorporate scientifically rigorous environmental–economic research into financial risk assessment for the world’s largest asset class, sovereign debt. We call on business schools and environmental scientists to accelerate collaboration and science-based financial innovation to deliver actionable insights that can improve the allocation of capital, create new financial instruments and products that support investments in adaptation as well as natural capital and biodiversity conservation, and meet globally agreed targets for biodiversity finance.

A key area for urgent collaboration is in the development of models that link environmental change to ecosystem service provision to economic outcomes. The GTAP-InVEST model on which our results are based is an important example. However, three key gaps remain. First, GTAP-InVEST facilitates the scenario analyses presented here, but does not support probabilistic statements about the likelihood of any given scenario coming to fruition. Thus, our results should be considered simulations rather than predictions. Second, while we have focused on biodiversity here, greater effort is needed to model and assess the combined, and potentially compounding effects of climate change and nature loss simultaneously. The Green-DICE model represents an important step, but does not yet provide global results at the same resolution as GTAP-InVEST^51–54^. Third, more work is needed to demonstrate the environmental benefits of conservation interventions at a macroscale so that these can be incorporated into financial risk assessments. Currently, countries using debt finance to support conservation and adaptation investments, may face downward pressure on credit ratings, as debt-to-GDP ratios increase and scarce resources are deployed in pursuit of nature-enhancing investments for public goods rather than traditionally productivity-enhancing investments for market returns. An important next step would be to combine GTAP-InVEST-type models with unified ‘pressures-and-conservation-impact’ (PACI) models^55^ that can incorporate the net effect of environmental change, accounting for both the negative consequences of nature loss and the positive benefits from conservation spending.

Finally, we call on academics from environmental science and finance to collaborate more closely with leading non-governmental organizations and established financial institutions—including credit rating agencies, the World Bank and the International Monetary Fund—to address shared concerns about the impact of nature loss on sovereign debt markets and the financial system more broadly. If there are two communities who ‘live or die’ by their ability to accurately identify and quantify future risks, they are environmental scientists and financial markets.

## Methods

Following recent innovations in the field of environmentally adjusted sovereign credit risk analysis^31,56^, a random forest classification model is trained on credit ratings data describing ratings issued by S&P and macroeconomic fundamentals to predict sovereign ratings. We then integrate forward-looking macroeconomic paths derived from GTAP-InVEST via a sovereign-level aggregation and indicator-adjustment layer, linking scenario outputs to the six ratings determinants in our prediction model (Supplementary Fig. 1). This implementation entails propagating nature-adjusted GDP (from GTAP-InVEST) into fiscal/external indicators via practitioner-based mappings, and embedding the resulting nature-adjusted GDP and nature-adjusted fiscal indicators into a ratings–PD–costs of borrowing pipeline.

We use the GTAP-InVEST earth–economy framework to link ecosystem service dynamics with macroeconomic outcomes. The model constitutes an original methodological contribution that integrates the GTAP computable general-equilibrium model of the world economy with the InVEST spatially explicit biophysical models. GTAP provides a consistent representation of global production, consumption and trade across 37 aggregated regions and 17 sectors, subdivided into 18 agro-ecological zones (341 AEZ-region units). InVEST is executed globally at this resolution to simulate changes in the provision of three ecosystem services—crop pollination, timber provision and marine fisheries—under alternative land-use and policy conditions. Timber productivity is represented through changes in forest biomass, crop pollination as a reduction in the ability of wild pollinators to pollinate crops, and marine fisheries as a reduction in the total catch biomass of marine fisheries.

Biophysical outputs from InVEST are converted into percentage productivity shocks for the corresponding GTAP sectors (agriculture, forestry and fisheries). These shocks are implemented as factor-neutral changes in total-factor productivity within the GTAP model, allowing ecosystem-service changes to propagate through production, trade and income linkages. The resulting general-equilibrium adjustments yield country-level measures of GDP, welfare and related macroeconomic indicators.

The most up-to-date GTAP-InVEST estimates impose two restrictions on our modelling: (1) reliance on three ecosystem services that can be modelled endogenously, and (2) the sample of countries for which the GTAP-InVEST framework provides data that can be reliably disaggregated to the national level, in our case 23 sovereign states.

While GTAP-InVEST constitutes a step change for integrated ecological–economic modelling, a range of assumptions and limitations remain. These arise from the nature of CGE modelling in general, the specific GTAP CGE model itself, the InVEST model, and issues encountered when linking models across multiple spatial scales. First, in its current form, GTAP-InVEST can only be used for comparative static analyses: it can compare a baseline equilibrium against future equilibria but is not a dynamic model that can endogenize environmental–economic–fiscal feedback loops over time. Second, the three ecosystem services included here are only a limited subset of the full spectrum of economically valuable services generated by nature. Third, and relatedly, many of the benefits generated by nature accrue to humans outside of formal markets and would not be captured in this analysis. Fourth, at present, the GTAP-InVEST model does not include a coupled, integrated financial sector. Lastly, CGE models entail a range of assumptions for key parameter values, including total factor productivity growth, substitution elasticities, representative household behaviour (in this case, Cobb–Douglas utility functions) and production frameworks (in this case, nested constant elasticities of substitution production functions). Accordingly, our results should be considered lower-bound estimates and interpreted as scenario simulations rather than predictions.

### Training the ratings model on historical data

Our parsimonious model uses six macroeconomic variables to predict sovereign credit ratings: GDP per capita, GDP growth, net general government debt/GDP, general government balance/GDP, narrow net external debt/CAR, and current account balance/GDP. We choose this six-indicator set because it aligns with the core economic (income level and growth), fiscal (debt stock and fiscal balance) and external (external leverage and external balance) pillars used in sovereign credit assessment, offers consistent cross-country coverage, and can be systematically adjusted under our biodiversity scenarios. GDP per capita and GDP growth are taken directly from the GTAP-InVEST outputs for each scenario. The four government-performance and external indicators are then updated using practitioner’s evidence^57^ on how GDP shortfalls relate to movements in these variables. Concretely, we fit low-order polynomial functions to the published relationships between disaster-induced GDP losses and each indicator, and we apply the nature-adjusted GDP outcomes to generate internally consistent, scenario-specific series by country. This design preserves predictive performance while avoiding ‘anchoring’ on determinants that cannot be adjusted on the basis of scientific evidence, and it keeps the scenario mechanics close to observable ratings practice.

We trained the algorithm using data on 113 countries from 2015 to 2020. Typically, in machine learning exercises it is best to include the largest possible sample. Despite longer data availability for most countries, we intentionally avoid the 2008–2009 global/Eurozone crises and the 2021–2023 pandemic years to reduce noise and maintain the highest possible predictive accuracy. We tested various periods, including 2004–2020 and 2012–2020, which are reported in Supplementary Section 3 with an extended time series. This process enables us to build statistical parameters within which we can begin to make predictions around how changes in our variables will influence credit ratings.

### Random forest model

Random forest models are classification algorithms. In our case, we classify sovereigns into investment-grade versus speculative-grade as a starting point, using six explanatory features. The model chooses the split that most reduces classification error—for example, if a GDP-per-capita threshold neatly separates investment-grade from speculative-grade issuers, that variable becomes the first split (the root node; Supplementary Fig. 2).

After the initial split, observations pass to the next node where the algorithm again selects the variable that most effectively reduces error. Iterating this yields clear boundaries between different levels of credit quality. Our implementation expands the simplified example to 20 rating classes and aggregates results over 2,000 trees. The estimated objects are the thresholds/boundaries separating categories. This approach offers two advantages: (1) each tree is trained on a perturbed version of the baseline data, producing largely uncorrelated predictions that we average; and (2) it mitigates overfitting, improving out-of-sample accuracy. Each split considers only a random subset of features, and the forest prediction is the average across all trees, which is typically more stable and reliable than a single model.

As illustrated in Supplementary Fig. 3 (left hand-side box), higher ln(GDP per capita) pushes ratings over successive thresholds, with pronounced nonlinearities: at very low income levels, further declines can leave predicted ratings unchanged, indicating other variables dominate in that range. GDP growth has strong effects over a narrower band: rising growth helps to move ratings into investment grade, but the effect fades beyond a point—potentially because very high growth may reflect post-shock rebounds where lingering vulnerabilities matter more. The remaining panels show analogous relationships for fiscal and external indicators.

### Variable importance

The importance of each variable for predicting ratings can be assessed by observing the percentage drop in *R*^2^ when each variable is permuted at random (Supplementary Fig. 4). This test indicates how much predictive accuracy would be affected if an individual variable from the set were replaced with a random value. While the ranking is illustrative and may vary by country, ln(GDP per capita) emerges as the dominant driver on average, followed by debt, fiscal balance and GDP growth.

To unpack country-level mechanics, Supplementary Fig. 5 decomposes predicted ratings for a range of economies by sequentially adding variables. Starting from the sample average predicted rating (11.155), incorporating additional information into the model ln(GDP per capita) lifts Canada by +5.428 notches (reflecting high income) but lowers China (given lower per-capita income). Proceeding through the remaining variables yields Canada’s final prediction of 19.672. This figure reinforces that contributions differ by country, underscoring heterogeneous variable importance.

### Benefits of random forest models

Random forests offer several benefits over econometric approaches for predicting sovereign ratings. First, we implement the above-described process thousands of times with slightly modified versions of the original dataset, each making use of a varied pool of the original six variables. This means that our prediction model will perform much better when presented with new data, adding precision to our estimates that no parametric approach such as regression can offer^58,59^.

Second, this approach enables us to model nonlinearities with greater ease^60^. Ratings data are peculiar, as they are discrete in nature (alphabetical ratings are translated into numerical scale such as the one we are using AAA = 20, AA+ = 19, SD = 1, with AAA representing the highest creditworthiness and SD the lowest). Incremental shifts through the rating scale do not represent equally meaningful changes in creditworthiness.

For instance, a 1-notch change at the top of the rating scale (for example, AAA to AA+) entails only a negligible increase in risk and default probability, whereas the same 1-notch change at the lower end of the scale (for example BB− to B+) can have substantial impacts on the cost and access to debt finance. Machine learning ultimately captures these dynamics. Moreover, ratings data exhibit distributional properties that can lead to bias in econometric modelling. There are often more observations at the top end of the ratings scale than across the rest of the rating categories. These features make linear modelling of credit ratings difficult and can lead to error. Finally, ratings are not purely quantitative assessments and reflect committee judgement and other qualitative considerations. Non-parametric methods help capture complex and nonlinear relationships between fundamentals and ratings more flexibly than traditional (parametric) approaches^61^. Therefore, using a methodology that can handle distributional properties, nonlinearities and qualitative components is essential.

### Adjusting ratings factors for environmental risk

Our ratings prediction model includes input variables that need to be adjusted to reflect natural capital and ecosystem service decline^56^. Shortfalls in GDP per capita and GDP growth for each scenario can be taken directly from GTAP-InVEST^2^. To adjust the remaining variables (net general government debt/GDP, general government balance/GDP, narrow net external debt/CAR, and current account balance/GDP), we exploit industry research conducted by S&P. S&P credit analysts were asked to assess how natural disasters such as earthquakes, tropical cyclones, floods and winter storms would affect GDP losses and government performance indicators^62^. Supplementary Fig. 8 shows data relating per capita GDP loss with analysts’ assessment of the impact on (the log of) net general debt to GDP. To produce the function that best describes this relationship, we fit polynomials of increasing order until no further improvement in fit could be found. We use this fitted model to relate GDP losses under each GTAP-InVEST scenario to each government performance variable.

One exception to this procedure is the production of results for Madagascar. S&P did not issue a rating for Madagascar during our 2015–2020 sample period. However, the country is included in the GTAP-InVEST results.

To avoid losing Madagascar from the sample, we used GDP per capita and growth data from the World Bank to estimate government performance variables based on the implied associations in the rest of the historical data. These were in turn used to estimate a starting point credit rating using the same random forest model described above. Incorporating the GTAP-InVEST results—as for any other country in the sample—produces biodiversity-adjusted credit ratings for Madagascar. For context, our simulated rating was 5.6, part way between B− (5) and B (6) (Supplementary Fig. 9). In April 2022, S&P issued Madagascar a B− rating. This exercise has no impact on the rest of the results and could be considered inconsequential, as Johnson et al.^2^ estimate losses exceeding 100% of Madagascar’s economy.

### Treatment of uncertainty

Our workflow links biodiversity loss to sovereign credit risk through a series of steps. Ecosystem service shocks generate scenario-conditioned macropaths for GDP levels and growth. These paths are then used to update fiscal and external indicators using practitioner-based mappings. The six adjusted inputs are finally translated into ratings and, from there, into PD and borrowing costs. Each stage introduces uncertainty (biophysical parameters, economic elasticities, sovereign-level harmonization and statistical prediction).

We report point estimates by scenario to match market practice in sovereign credit assessment. Credit ratings are single alphanumeric opinions on future default risk. Committees and market users usually communicate central judgements rather than full probability distributions. Practitioners such as S&P’s^57,62^ estimate how extreme weather events would affect sovereign creditworthiness, expressed as a change in rating notches relative to a base case rather than as a full distribution of outcomes. Our presentation mirrors this practice, while acknowledging that parameter and model uncertainty is present at several stages.

Although sampling over the full uncertainty space would be ideal, the principles guiding this study emphasize staying close to how rating agencies handle uncertainty in practice. Our team includes a former Global Sovereign Chief Rating Officer at S&P who helped build the sovereign rating methodology and sat on hundreds of committees, including those that issued ratings for countries examined here. This experience informs our choice of presentation.

We address uncertainty in three ways. First, we use clearly defined scenarios that function as stress tests. These bracket plausible outcomes under large ecological shocks rather than assigning explicit probabilities. Second, when mapping nature-induced GDP shortfalls to fiscal and external indicators, we fit low-order polynomial functions to practitioner evidence. Third, in the ratings step, we report out-of-sample diagnostics such as accuracy within ±1 or ±2 notches, and we rely on the internal resampling of the random forest procedure to gauge predictive dispersion without imposing a parametric error structure.

Sampling over the full uncertainty space remains an important issue and is a priority for future work. It is outside the scope of this study.

### Calculating default probabilities and additional cost of debt

Once we obtain the biodiversity-adjusted credit ratings, we can translate them into additional costs of borrowing of sovereigns and corporates. We first converted S&P’s sovereign ratings to a 20-notch scale (Supplementary Fig. 9).

To calculate the additional cost of borrowing associated with nature-driven sovereign downgrades, we estimate the additional premium over the risk-free rate that would be associated with a sovereign at its baseline rating. With the newly estimated, nature-driven sovereign credit rating, we re-establish the additional premium. The difference between these two values is the additional cost of debt. For example, China, which has a baseline estimated rating of 15, would have a spread of 1.05%. With a nature-driven rating of 9.5, the new spread would be 2.81. The increase in the cost of borrowing is therefore 1.76% (2.81 − 1.05 = 1.76). The cost of borrowing figures for the rating categories are established by taking the median option-adjusted spread for each rating category and interpolating as per Supplementary Fig. 10.

A similar process is conducted using data from S&P Global to estimate the increase in the 10-year PD. Supplementary Fig. 10 reveals the model used to estimate the spread increase in the cost of debt (Supplementary Fig. 10a) and the increase in the PD, respectively (Supplementary Fig. 10b). Our model for estimating the increase in cost of debt and the PD is given by the parameters from a cubic and quintic regression, respectively.

### Calculating additional debt burden from nature loss for the median citizen

Figure 3 (right) was calculated by obtaining the median income or consumption from Our World in Data^63^. For each country, we take the most recent year observation to calculate the per capita increase in the cost of debt (associated with the nature-driven downgrade) using the population data from the corresponding year. We then divide this number by the median income or consumption (multiplied by 365, as the data are daily) for the same year to obtain the proportion of the nature-driven borrowing cost that represents the median person’s annual income.

### Reporting summary

Further information on research design is available in the Nature Portfolio Reporting Summary linked to this article.

## Supplementary information

Supplementary Information Supplementary Figs. 1–10 and Tables 1 and 2.

Reporting Summary

Peer Review File

## Data Availability

Data were hand-collected from publicly available sources. Data on sovereign credit ratings and ratings factors are sourced from S&P Ratings Direct database and S&P Sovereign Risk Indicators public depository. Data on GDP impacts associated with ecosystem service losses are taken from the GTAP-InVEST model^2^. Median after-tax (that is, disposable income after all taxes are paid) income is sourced from Our World in Data^63^. The full replication package, including code and data, is available via GitHub at https://github.com/mattdburke/nature-credit.

## Code availability

The full replication package, including code and data, is available via GitHub at https://github.com/mattdburke/nature-credit. The software used for the analysis in this article was version 4 of R. A variety of different R packages were used to support data cleaning and compiling figures and graphs. All of these packages are standard and listed in the GitHub repository, along with a script in the main file that automatically loads or downloads these packages.
