# Supplementary material for: Biodiversity loss will decrease the future creditworthiness of nations
Source: Nat Ecol Evol. 2026 Jun 5;10(8):1511–20. doi: 10.1038/s41559-026-03081-7 (PMC13441964; doi:10.1038/s41559-026-03081-7)
Supplement: Supplementary file 1 [file 41559_2026_3081_MOESM1_ESM.pdf]

nature ecology & evolution

Supplementary information https://doi.org/10.1038/s41559-026-03081-7

Biodiversity loss will decrease the future creditworthiness of nations

In the format provided by the authors and unedited

1. How the Random Forest Model Operates

Supplementary Figure 1 Model building and prediction process

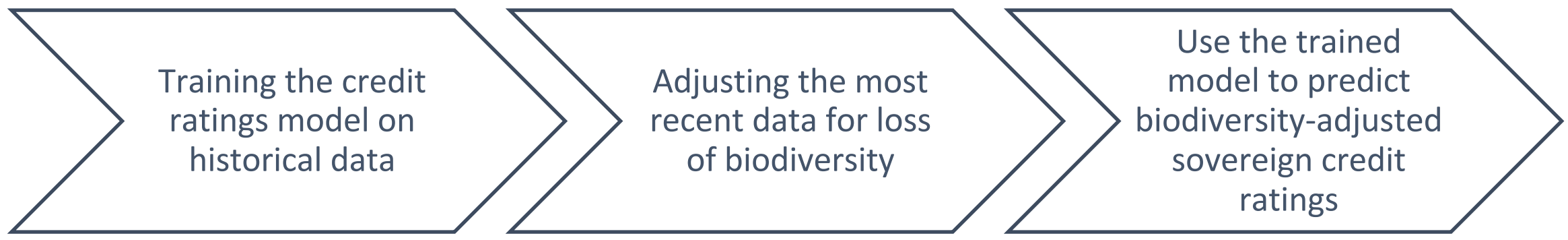

Process for incorporating biodiversity and ecosystem service loss into sovereign credit ratings. First, we train a random forest classification model on historical credit ratings data to create a ratings prediction algorithm. Next, we feed the ratings prediction algorithm created in step 1 forward-looking projections of macroeconomic outcomes under a range of scenarios. Finally, we use the trained model to simulate biodiversity-adjusted sovereign ratings.

Supplementary Figure 2: Random-forest classification workflow

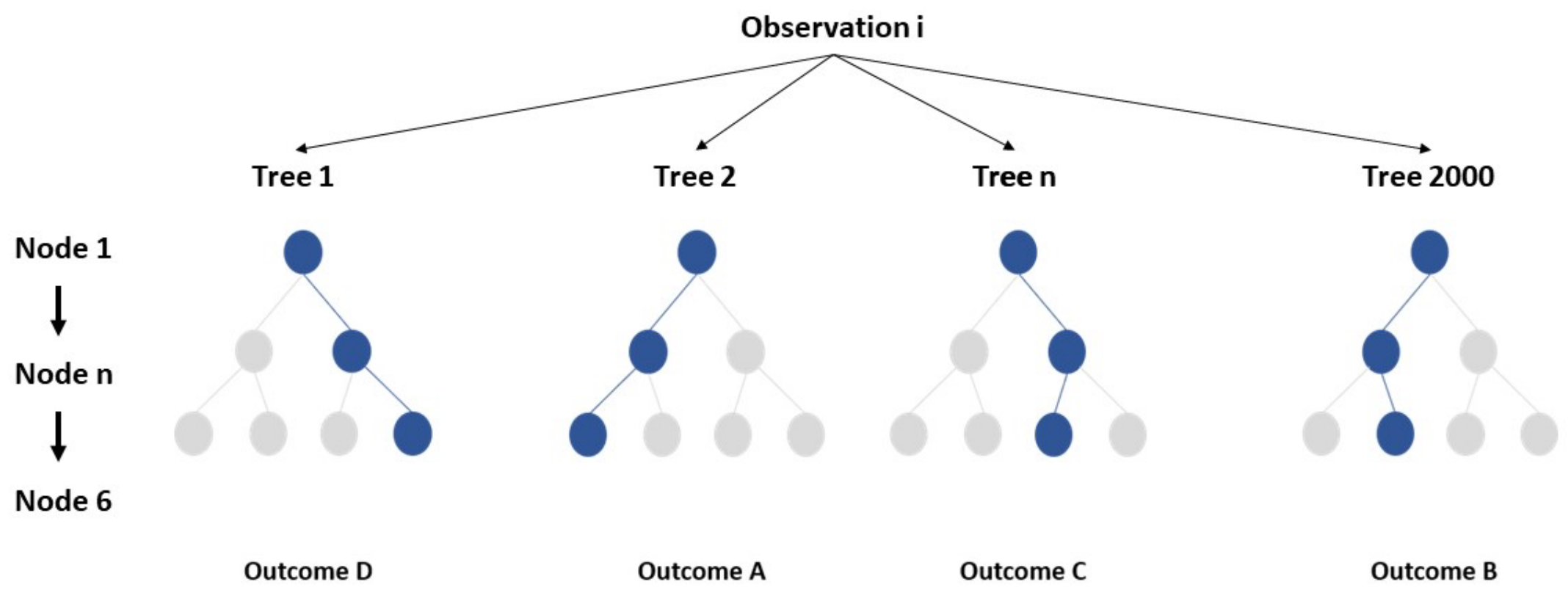

Supplementary Figure 2 sketches the simplified workflow used to predict sovereign ratings. For each country we grow 2,000 trees with 6 nodes each. The schematic shows four possible outcomes, whereas the full model classifies into 20 categories corresponding to the 20-notch ratings scale.

2. How each variable operates within the model.

Supplementary Figure 3 Partial effects of rating drivers in the random-forest model

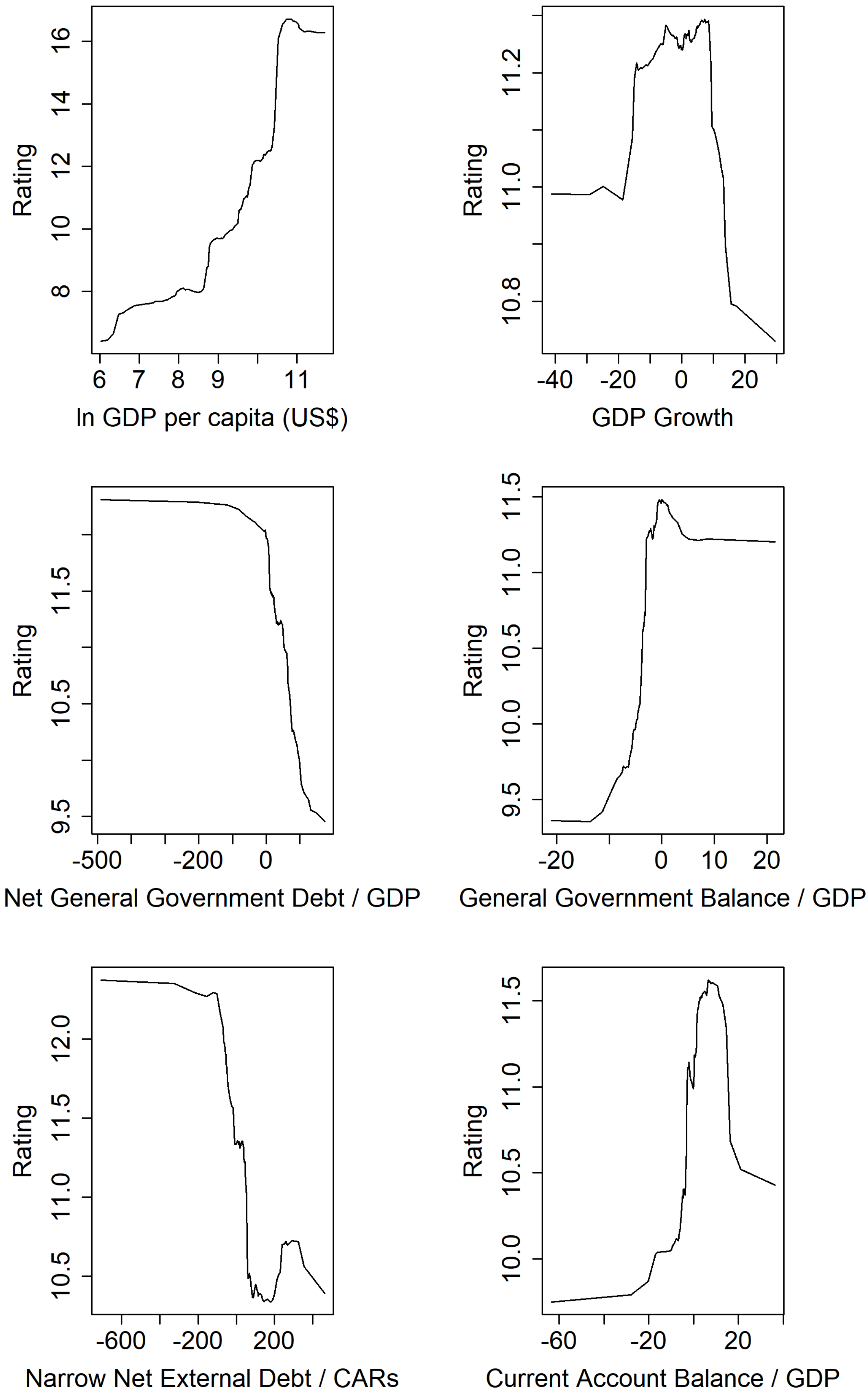

Supplementary Figure 3 shows the relative importance of six variables in the model. The x-axis shows partial effects for ln(GDP per capita), GDP growth, net general government debt/GDP, general government balance/GDP, narrow net external debt/CARs, and current account balance/GDP; the y-axis indicates the implied rating level, conveying both direction and relative importance.

Supplementary Figure 4: Relative importance of variables in predicting ratings

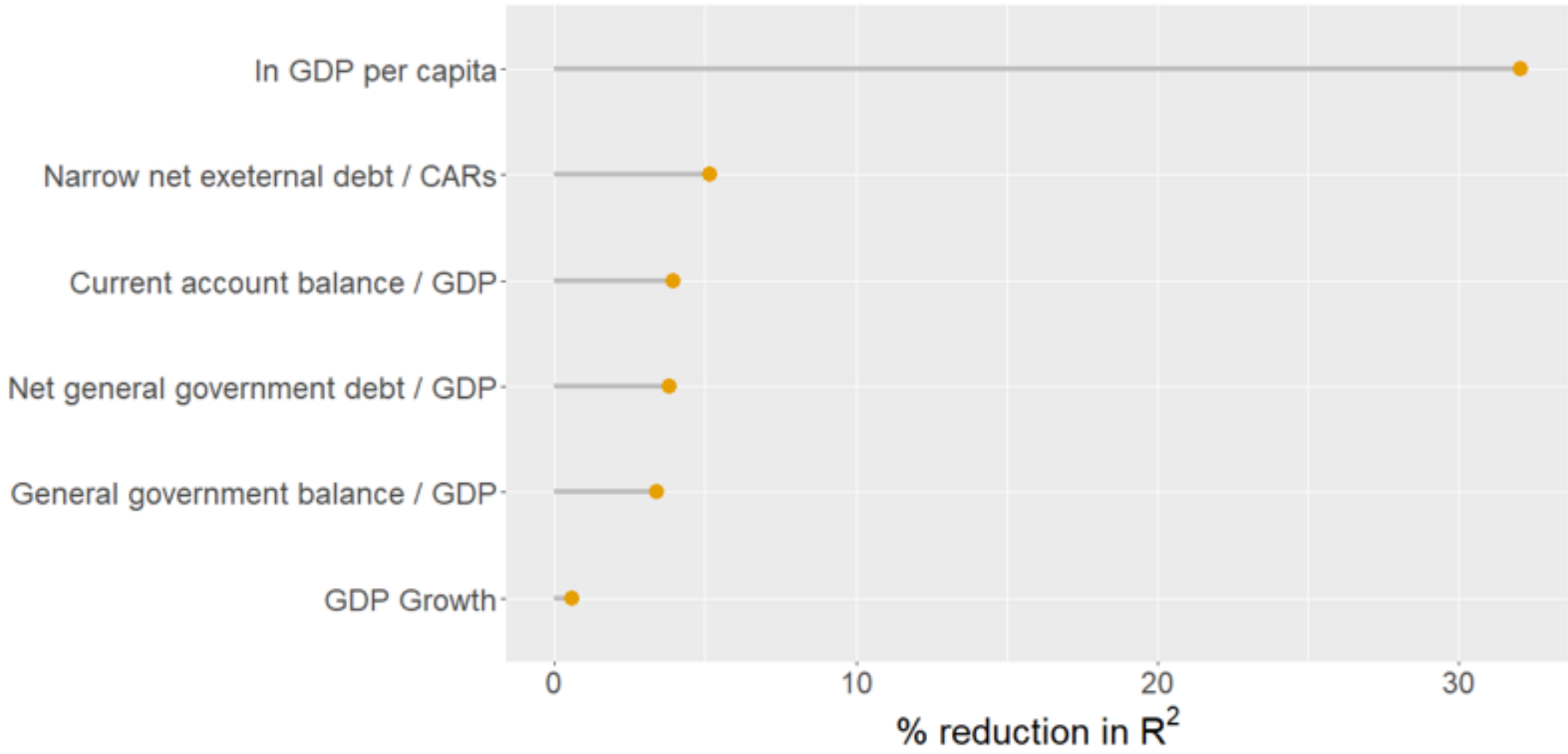

Supplementary Figure 4 indicates the relative importance of variables in the ratings prediction model. It shows the percentage drop in R^2 which would be observed after replacing any individual variables with random values. The sample comprises 639 ratings across 109 countries during 2015–2020.

Supplementary Figure 5 Ratings projections (selected economies)

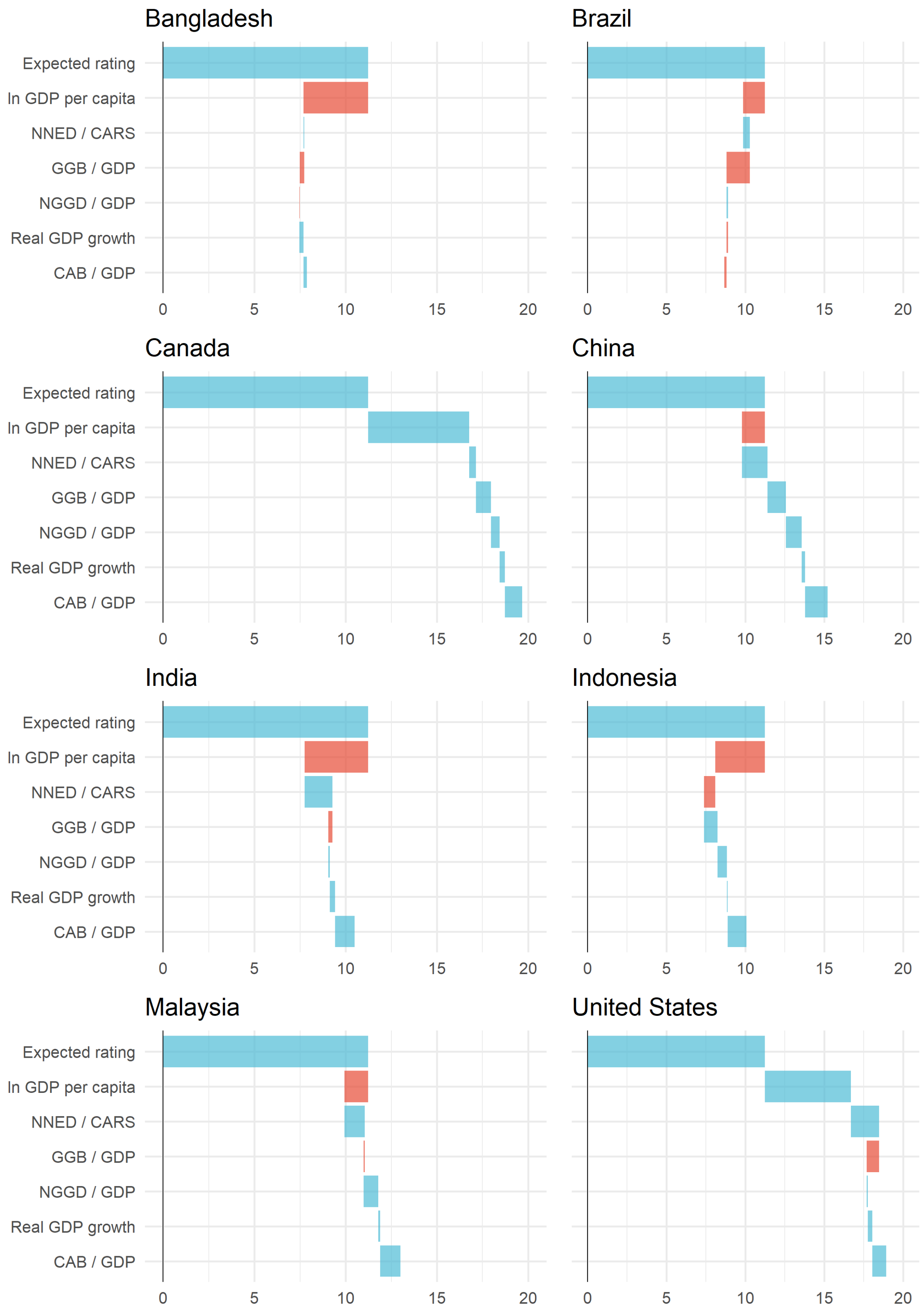

Supplementary Figure 5 shows how each ratings factor in the model affects the predicted rating for selected countries. On first pass, the 'best guess' prediction for the rating of any country is simply the

average rating across the sample. For each country, the figure illustrates how adding the next variable shifts the predicted sovereign rating away from the sample-average baseline (11.155). The sign and magnitude of contributions from each variable vary by country. For instance, ln GDP per capita contributes positively to Canada's rating (blue bar) but negatively to China's rating (red bar).

To unpack country-level mechanics, Supplementary Figure 5 decomposes predicted ratings for a range of economies by sequentially adding variables. Starting from the sample average predicted rating (11.155), incorporating additional information into the model ln(GDP per capita) lifts Canada by +5.428 notches (reflecting high income) but lowers China (given lower per-capita income). Proceeding through the remaining variables yields Canada's final prediction of 19.672. This figure reinforces that contributions differ by country, underscoring heterogeneous variable importance.

3. Predictive accuracy of the ratings simulation model

Supplementary Table 1 evaluates how well our ratings simulations model matches published S&P sovereign ratings prior to adding climate-related adjustments. Rows report the share of cases within N notches (N=0–3). Columns present the full sample (col. 3), an 80/20 out-of-sample split for all countries (col. 4), and an 80/20 out-of-sample split limited to investment-grade issuers (col. 5).

Supplementary Table 1 Validation metrics for the ratings predictor

		**Whole sample**	**Out of sample 80/20% split**	**Investment grade only. Out of sample 80/20%**
% predicted within n notches	N = 0	68.01	34.26	45.45
	N = 1	96.43	79.63	87.27
	N = 2	99.84	94.44	94.55
	N = 3	-	98.15	-
Observations		644	536 / 108	270 / 55
Countries		109	109 / 108	60 / 55

Notes: Supplementary Table 1 reports the percentage of predictions within 0–3 notches for the whole sample, an 80/20 out-of-sample split, and investment-grade-only 80/20 out-of-sample tests. Coverage: S&P ratings, 2015–2020.

In our benchmark out-of-sample setting (col. 4), exact matches occur 34.26% of the time, and over 94% of predictions are within two notches. Prior work suggests excluding recent defaulters can raise accuracy. Restricting to investment-grade countries (col. 5) slightly increases exact matches but reduces the within-two-notches rate—likely because the training sample shrinks by nearly half. Excluding lower-rated countries could also bias results if climate vulnerability correlates with low ratings through agriculture exposure, infrastructure resilience, or governance quality. We therefore retain all 109 countries and train on S&P ratings from 2015–2020.

Supplementary Figure 6 visualises the out-of-sample performance reported in Table 3 (col. 4). The 45-degree line marks perfect agreement between predicted and observed ratings. Each country appears as a point at its predicted value, alongside its prediction

error. Out-of-sample, the model exactly matches the published rating in 34.26% of cases and lands within two notches for over 94%. Uncertainty is quantified using jackknife standard errors following Wager et al. (2014). Model error reflects variability inherent to the random-forest estimator: every one of the 2,000 trees produces a rating, and the whiskers for each observation span the range of those tree-level predictions.

Supplementary Figure 6 Model performance on out-of-sample data (visual summary)

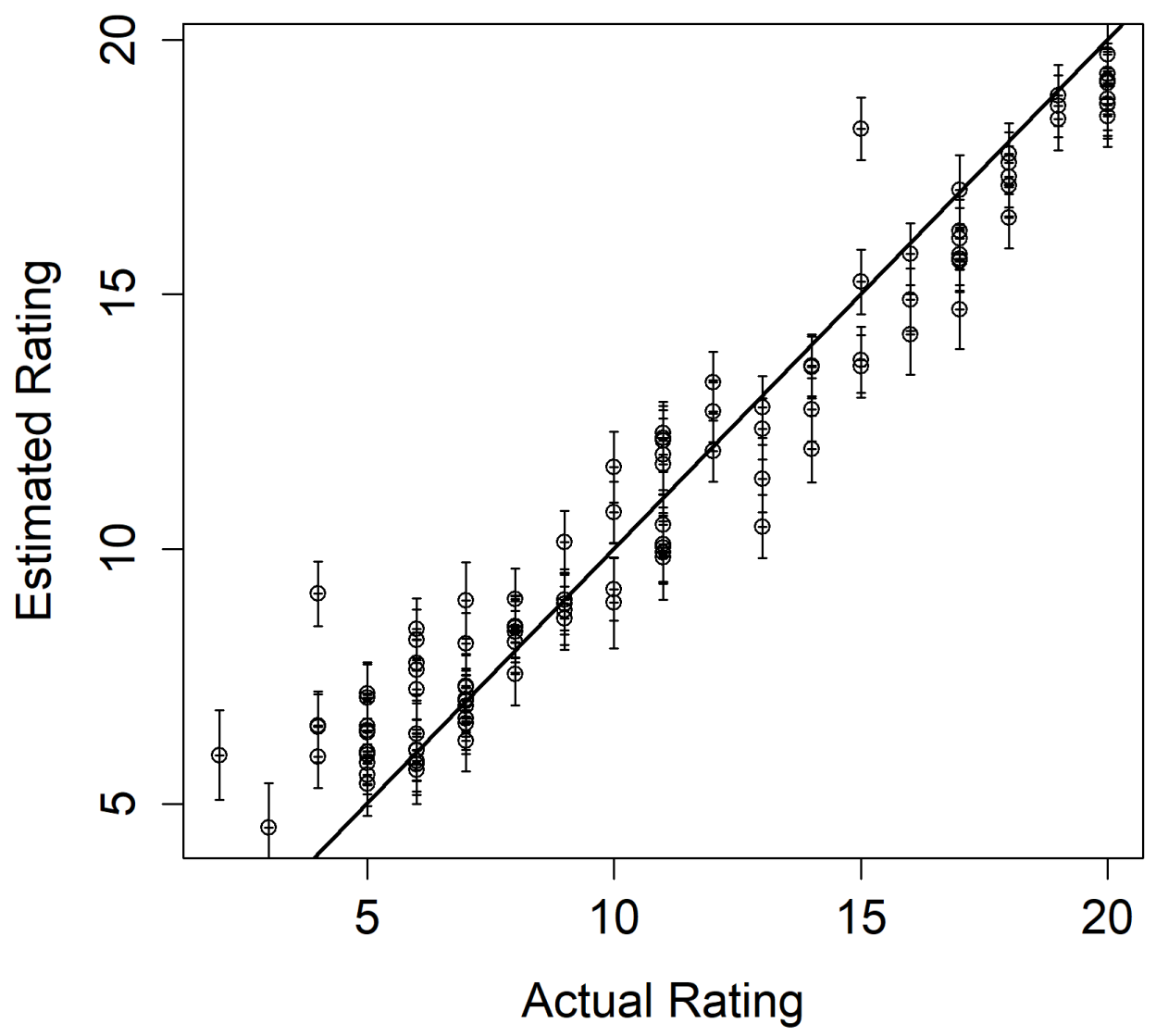

Supplementary Figure 6 plots observed 2020 ratings (x-axis) against out-of-sample predictions (y-axis). The 45-degree line denotes perfect accuracy. Data are presented as mean values. Whiskers indicate the range of tree-level predictions across the 2,000 trees.

Supplementary Figure 7 maps how predictive accuracy varies across countries. High accuracy is not concentrated in any specific region, climate zone, country size, development level, or political system. Exact matches occur for a wide mix of sovereigns—including Brazil, Finland, Uganda, Germany, Honduras, and Mongolia—while most G20 members are predicted within one notch. The weakest fits are for Argentina, Colombia, Ecuador, and Iraq, which is consistent with their histories of crisis, default, conflict, or political instability. We do not incorporate governance variables directly, as we lack robust, quantifiable estimates of nature loss impacts on those indicators.

Supplementary Figure 7 Spatial pattern of out-of-sample accuracy

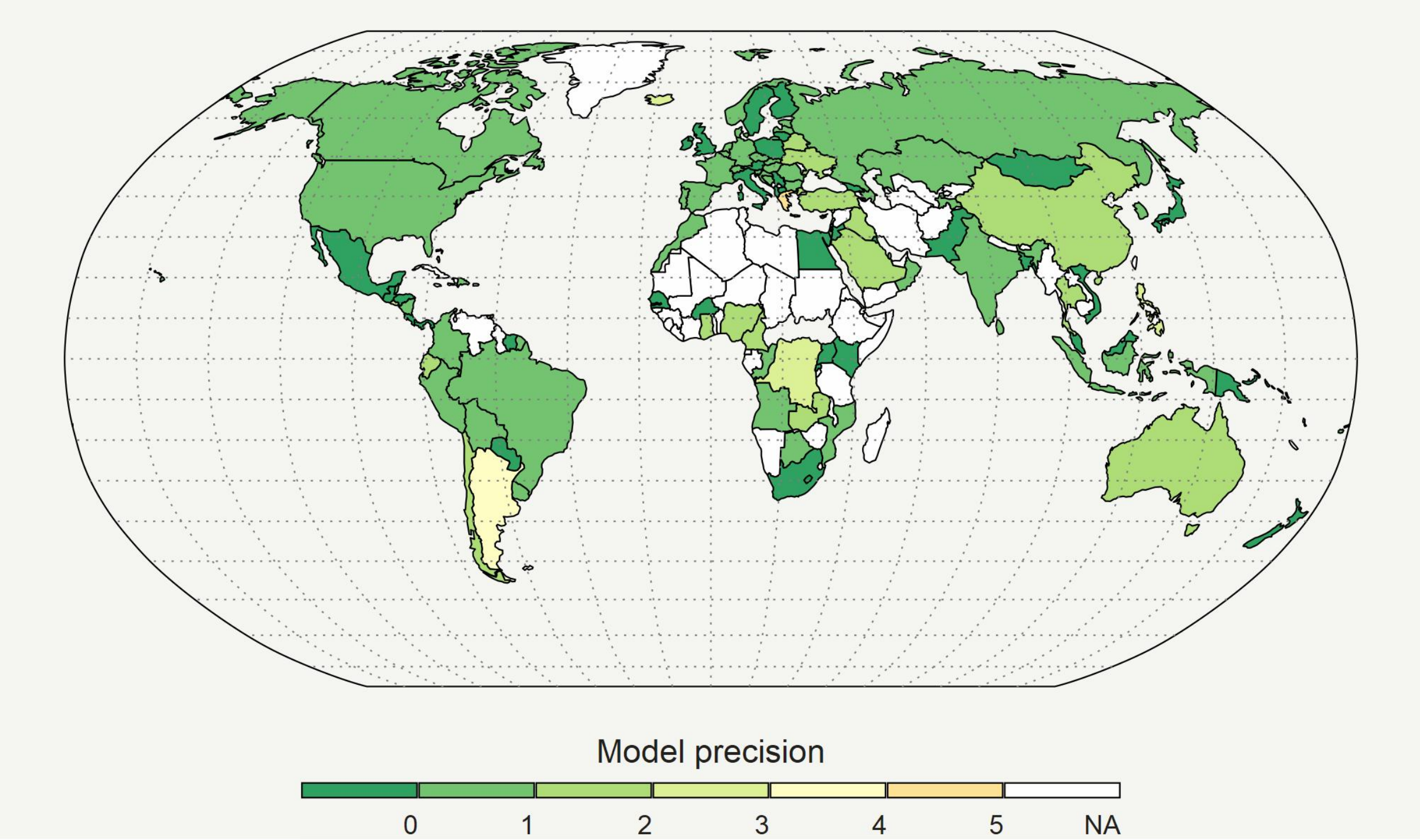

Supplementary Figure 7 maps the model's out-of-sample accuracy. Strong performance spans diverse regions, sizes, income levels, political systems, and climates. Countries without S&P ratings are not evaluated. Model precision is indicated by colour. Dark green indicates a perfect match between our predicted rating and the observed rating issued by S&P Global. Lighter colours reflect the absolute value of the deviation in notches (on S&P Global's 20-notch scale) between predicted and observed ratings.

Predictive accuracy with an extended time series

Tripling the time window lifts the sample size from 644 observations in the baseline to 1,590 in the longer panel. We retain the identical six predictors and the same set of 109 countries. Table SI.2 contrasts the baseline configuration (cols. 2–4) with the extended-series version (cols. 5–7). With the longer window, exact matches decline for in-sample, out-of-sample, and investment-grade out-of-sample tests. Because the model is stochastic, swings of this magnitude appear across repeated runs. Notably, expanding the timespan has asymmetric effects depending on the test universe: for all 109 countries (cols. 2 vs. 5), accuracy falls across all bands, whereas for investment-grade-only tests (cols. 4 vs. 7) exact matches dip slightly but the shares within 1, 2, and 3 notches improve.

At first glance, this seems at odds with the idea that more data should help. But sovereign ratings have a practical "shelf life" of roughly 5–10 years, as underlying economic and political conditions evolve. Incorporating much older observations can therefore dilute signal. More importantly, the extended window spans the run-up to, peak of, and aftermath from the 2008 global financial crisis—a period of unusual volatility in fiscal metrics and ratings. That turbulence likely injects noise that outweighs any gain in predictive content. Importantly, extending the series does not materially change the

simulated climate-impact results on ratings. For these reasons, our baseline focuses on ratings from 2015 to 2020.

Supplementary Table 2. Predictive performance under an extended sample period

Rating range	Our results (2015 – 2020)			Extended sample (2004 – 2020)		
	Whole Sample	Out of sample (80/20 split)	Investment Grade Out of Sample (80/20)	Whole Sample	Out of sample (80/20 split)	Investment Grade Out of Sample (80/20)
Exact match	68.01%	34.26%	45.45%	57.17	23.12	30.11
1 notch	96.43%	79.63%	87.27%	94.03	61.25	72.73
2 notch	99.84%	94.44%	94.55%	98.36	85.00	94.89
3 notch	-	98.15%	-	99.56	92.81	98.86
Observations	644	536/108	270/55	1590	1270/320	710/176
# of variables	6	6	6	6	6	6
Countries	109	109 / 108	60/55	109	109/109	62/62

Notes: Columns 2–4 replicate Table SI.1 (2015–2020). Columns 5–7 use 2004–2020. Results reflect the same six-variable model and country coverage.

4. Adjusting Ratings Factors for Environmental Change

Supplementary Figure 8: Modelling of the effect of GDP loss on government performance variables

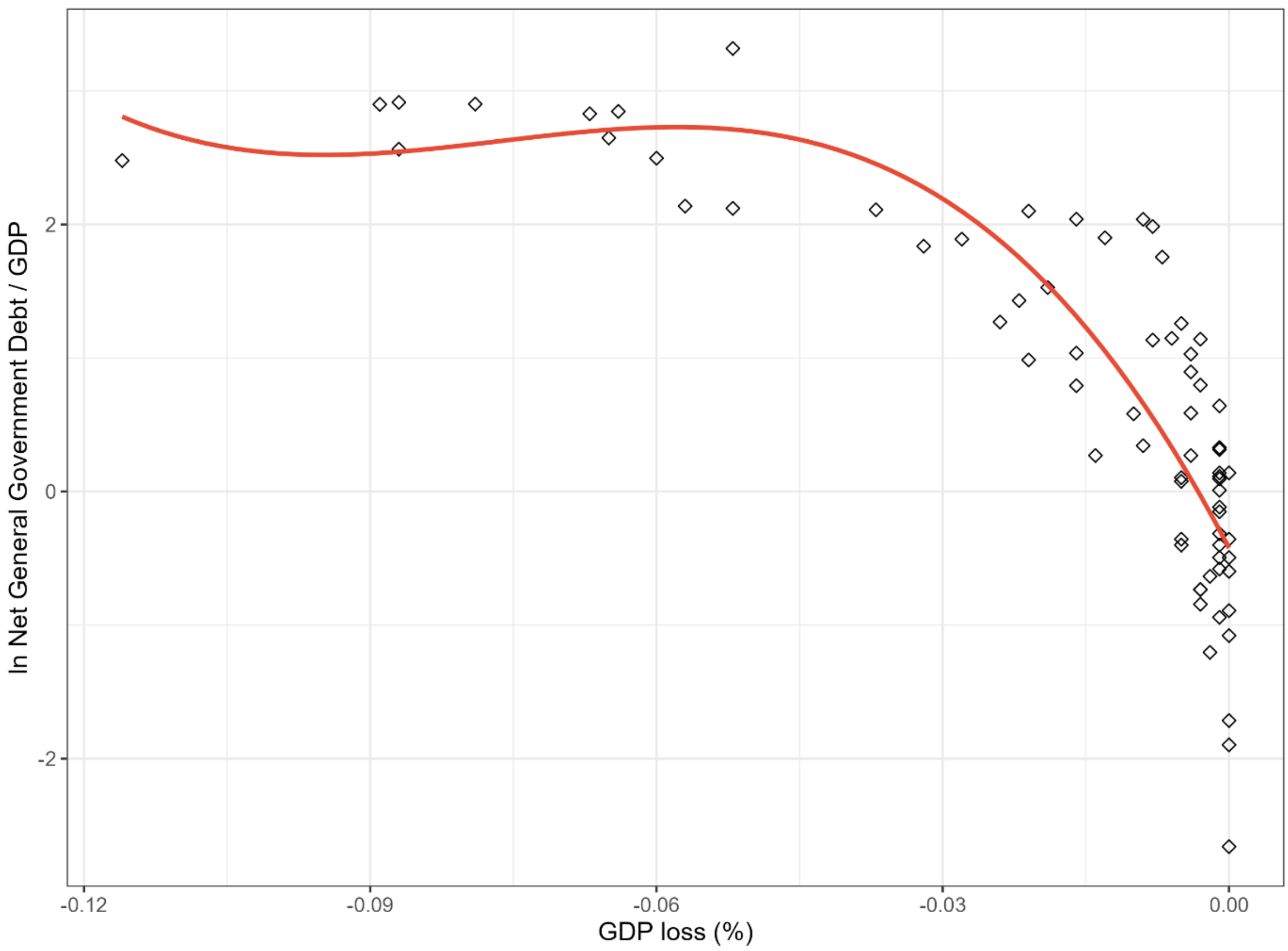

Source: Analysis based on data from S&P (2015)

Supplementary Figure 8 depicts the process for adjusting government performance indicators to nature loss (with an example of Net Government Debt to GDP). S&P[61] provides estimates of how natural disasters would affect GDP loss and other government performance indicators. We plot these estimates between GDP per capita loss and each indicator and fit a function that best describes this relationship. This function is then used to adjust each indicator for the GDP losses due to nature loss under various scenarios.

5. S&P Global's Sovereign Rating Scale

Supplementary Figure 9: Conversion of alphabetical S&P's sovereign ratings to 20-notch scale

Long-term Foreign currency issuer rating symbol	Numerical rating	Rating grade	
S&P			
AAA	20	Prime high grade	Investment grade
AA+	19	High grade	
AA	18		
AA-	17		
A+	16	Upper medium grade	
A	15		
A-	14		
BBB+	13	Lower medium grade	
BBB	12		
BBB-	11		
BB+	10	Speculative	Non- investment grade
BB	9		
BB-	8		
B+	7	Highly speculative	
B	6		
B-	5		
CCC+	4	Substantial risks	
CCC	3		
CCC-	2		
CC	1	Extremely speculative	
C	1		
D/SD	1	In default	

Supplementary Figure 9 presents S&P Global's long-term foreign currency credit ratings scale. On the 20-notch scale, AAA is the highest possible rating. The cut-off between investment grade and non-investment grade is between BBB- (11) and BB+ (10).

6. Estimating spreads and default probabilities arising from ratings changes.

Supplementary Figure 10: Estimating spreads and default probabilities from ratings changes

A

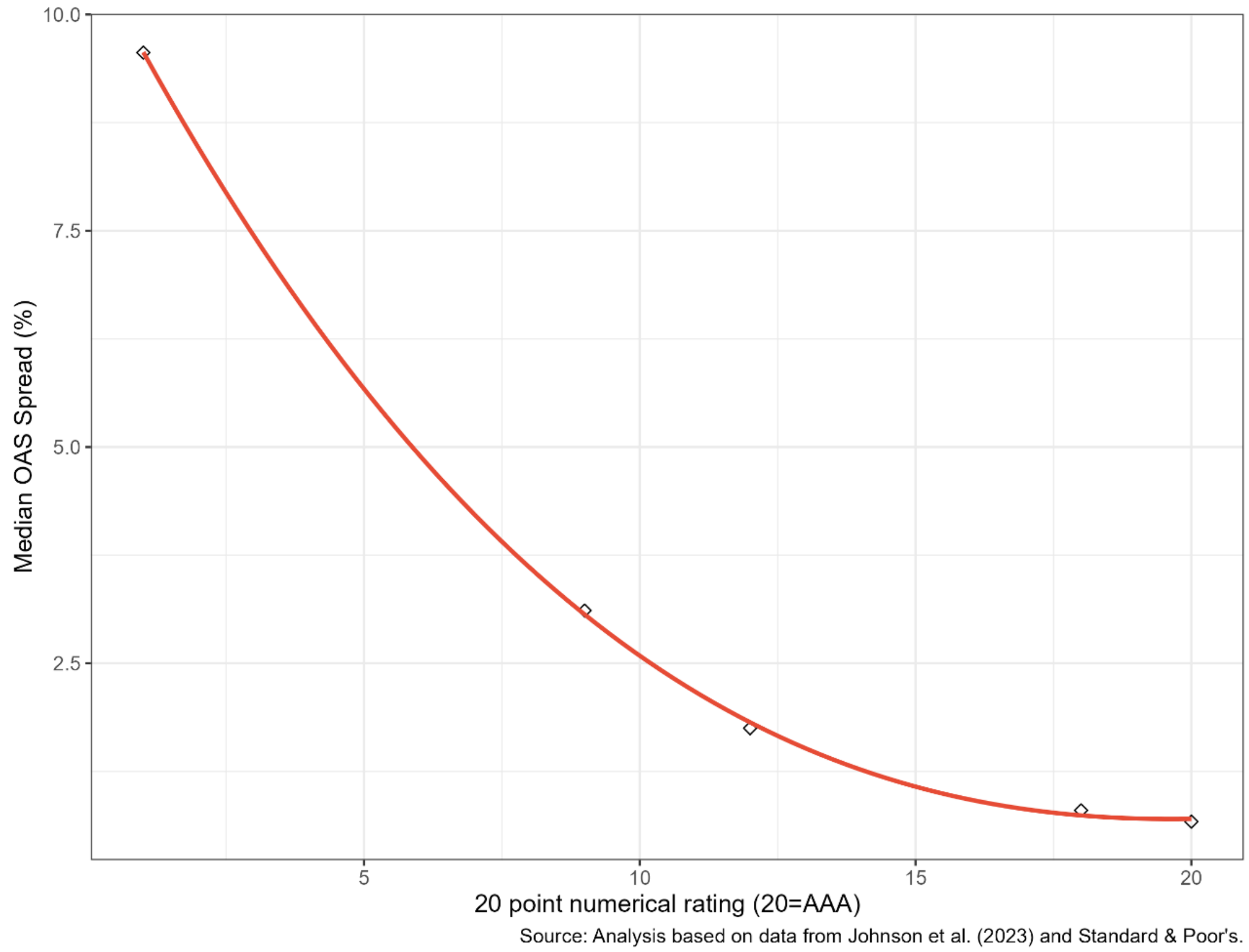

B

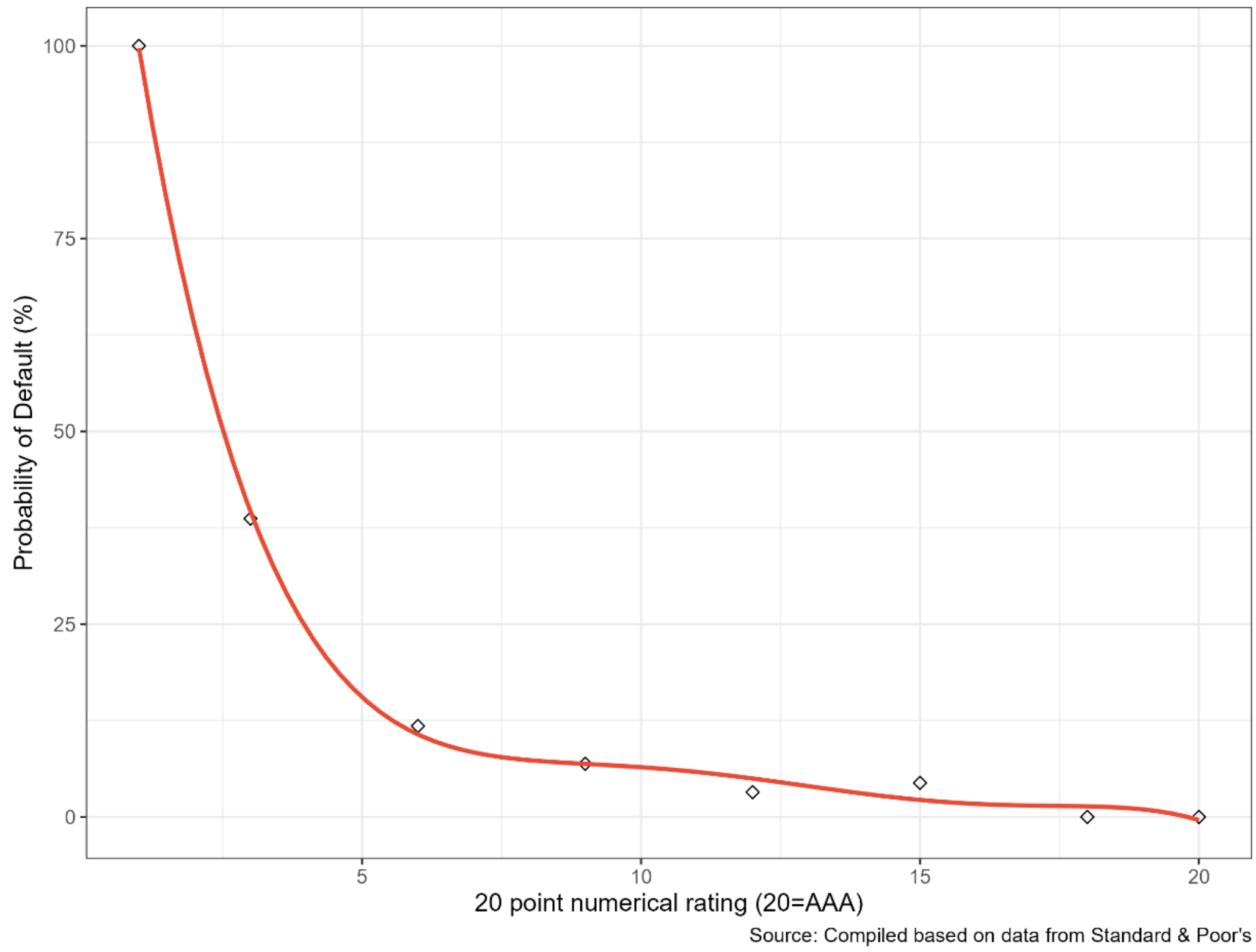

Supplementary Figure 10 reveals the model used to estimate the spread increase in the cost of debt (Panel A) and the increase in the probability of default respectively (Panel B). Our model for estimating the increase in cost of debt and the probability of default is given by the parameters from a cubic and quintic regression respectively.
