# Supplementary material for: Biodiversity loss will decrease the future creditworthiness of nations
Source: Nat Ecol Evol. 2026 Jun 5;10(8):1511–20. doi: 10.1038/s41559-026-03081-7 (PMC13441964; doi:10.1038/s41559-026-03081-7)
Supplement: Supplementary file 3 [file 41559_2026_3081_MOESM3_ESM.pdf]

nature portfolio

Peer Review File

Biodiversity loss will decrease the future creditworthiness of nations

Corresponding Author: Professor Matthew Agarwala

This file contains all editorial decision letters in order by version, followed by all author rebuttals in order by version.

Version 0:

Reviewer comments:

Reviewer #1

(Remarks to the Author)
Thanks to the authors for this well-written, clear, and important paper.

A few questions/comments, to cut to the chase:
1. The partial ecosystem collapse (PEC): why was a 90% reduction chosen? What rationale for this percentage? I kept wondering what the likelihood of a PEC would be, and what conditions might lead to it. It seems to me that this is crucial information missing from the paper. At present it feels like this 90% is "picked out of a hat" and lacking context. Without a good rationale – linked to some justification - I would even suggest that the PEC scenario should be excluded from the paper.

2.On page 6 the authors point to the non-linear relationship between changes in GDP vs credit ratings. This non-linearity is not explained, nor is the differences between the different countries.

3.Do the authors see any connections between this research and the literature on financial subordination (e.g. https://academic.oup.com/cje/article-abstract/46/4/679/6598707; https://www.tandfonline.com/doi/full/10.1080/09692290.2022.2098359). It seems to me not-insignificant that the countries most impacted are often "subordinated" in the wider global political economy. There might be more insights to draw out here, particularly related to Article 20 of the CBD, which is essentially a common but differentiated responsibility clause, but also connections to development financing questions and the wider efforts to bring into being a multilateral debt mechanism.

4.Is this paper really about biodiversity? As far as I can see, based on the limitations of the INVEST model, this is about ecosystem services, and a very (very) narrow three services. I think it is important to recognize that this is a proxy for biodiversity, but a very imperfect one, particularly for the Nature: Ecology and Evolution audience (and for a paper that self-identifies as being closer to ecology and science than other nature risk work). I would like to see the authors say a bit more about this limited ecological range, particularly as it relates to biodiversity. While the authors suggest that this narrow range of ES modeled means a likely underestimation of impact, I am thinking that a whole lot of biodiversity can be lost before one might see impacts on financial assets

(Remarks on code availability)

Reviewer #2

(Remarks to the Author)

The paper aims to analyse the impact of biodiversity loss on sovereign creditworthiness using S&P ratings and biodiversity loss impacts derived from GTAP–InVEST.
The assumptions are that sovereign ratings are currently not pricing biodiversity loss in sovereign ratings, and that biodiversity loss is expected to worsen country's economic competitiveness, leading to an increase sovereign debt issuance needed to finance investments in nature restoration. A worsening in sovereign ratings would make it more expensive for

countries to borrow on the market, increasing sovereign debt even further.

Genereal comments

The topic is of interest for the finance community as shown by growing research (Garel et al. 2024, Flammer et al. 2025) and a recent special issue on Biodiversity finance (Giglio et al. 2025) published in Review of Finance, and another one published in Ecological Economics (the latter one edited by some of the coauthors of this paper, see Guo et al. 2025). While being of interest, the focus of the paper is not particularly novel, nor is the approach, that includes recent contributions of the authors for the introduction of climate risk into sovereign ratings (Klusak et al. 2023).
In addition, the implementation of the analysis faces several methodological limitations that are not properly discussed nor addressed.
While I am very sympathetic with the authors' view that governments, central banks, financial regulators, and international financial institutions should not ignore nature-related financial risk, I am not persuaded that the approach proposed here would help them in this process.

Specific comments

There are some well known limitations to the inputs the rating model uses to embed biodiversity risk coming from the GTAP–InVEST model, which is a Computable General Equilibrium model (CGE) and are not properly discussed. These include CGE assumptions of linearity of shock, rationale expectations, single optimal intertemporal equilibrium, and lack of finance and finance-economy feedback that are well known in the field and lead to a misestimation of the impact of even large shocks on GDP.

The use of a coupled CGE–nature framework (GTAP–InVEST) to generate economy-wide shocks that propagate through global trade linkages before being fed into the ratings model is not novel and nature VaR estimates already exist at more granular level (see Ranger et al. 2024 for the UK).

The novelty regarding the integration of nature into macro-financial risk analytics should be revised at the light of the growing literature in the field combining ENCORE dependency/exposure mappings with input–output or loan-book data layers and sector–ecosystem service linkages (see NGFS's Green Scorpion, Ranger et al. 2023) and integration of the NVaR into a macroeconomic model with similar features (Nigem, see Ranger et al. 2024).

Scenarios play a key role in financial risk assessment and ratings adjustment. Yet here the scenarios are built exogenously, and there is no real scenario design. Also, it is unclear to what extent if any a main driver of the magnitude of the shock in the economy - i.e. the correlation of ecosystem dimensions and shocks, and their compounding - is modelled and analysed. This is a main limitation as nature and climate risks compound, among themselves and with climate risks (Ranger et al. 2024). Correlation and compound may lead to much larger economic impacts of biodiversity loss results, and through that on the fundamentals used for the financial risk analysis. These limitations are overlooked in the analysis and yet are important to understand the relevance of the shocks in the economy and their transmission to sovereigns.

The long term focus of the analysis via the S&P long-term foreign-currency sovereign ratings may be misleading. Investment decisions have a much shorter time horizon, as finance expectations may adjust abruptly as a result of new information on biodiversity risk exposure of the country, and lead to an adjustment of the credit rating well before the actual biodiversity loss occurs. Thus, long term foreign-currency sovereign ratings may not be the most relevant one.

The results show large impacts, in particular for still high GDP growth countries China and India. I have some concerns regarding the numbers. Ecosystems do not deplete all at once. IPBES (2022) shows that biodiversity degradation is already occurring, and yet the impact on the economy is still unclear. However, we can assume that if main ecosystems resources are missing, in times of growing geopolitical tension and trade barriers that limit import of key raw materials, countries most affected would start to act to build resilience to such losses, to prevent the risk of full collapse. Before a country goes to default, the central bank may also intervene (see the management of the Greek debt crisis last decade). Nevertheless, the analysis does not provide a simulation of the endogenous fiscal policy and monetary policy response that may be well triggered by ecosystem degradation, well before a collapse occurs.

If countries do not do anything, in the case of a partial ecosystem collapse, the related economic impacts would show up in the shorter term. Investors would look at countries' declining fundamentals, and prepare to adjust for that in the rating.An economic downturn would be transmitted to all financial contracts and securities, most likely starting from stocks as a result of worsening firms' performance. Only in case of expectations of a country getting close to default, its sovereign creditworthiness and ratings would massively adjust.
Thus, would a partial ecosystem collapse realise, the role of ratings may be the last ones occurring on the pipeline and the last to care as the economy would have already collapsed.

References

Flammer, C., Giroux, T., & Heal, G. M. (2025). Biodiversity finance. Journal of Financial Economics, 164, 103987.

Garel, A., Romec, A., Sautner, Z., & Wagner, A. F. (2024). Do investors care about biodiversity?. Review of Finance, 28(4),

1151-1186.

Giglio, S., Kuchler, T., Stroebel, J., & Zeng, X. (2025). Biodiversity risk. Review of Finance, rfaf063.

Guo, C., Agarwala, M., FREEMAN, M. C., Groom, B., & McKenzie, E. (2025). Introduction to the special issue:'Biodiversity and finance: Risk, disclosure and double materiality'. Ecological Economics, 240, 108798.

Klusak, P., Agarwala, M., Burke, M., Kraemer, M., & Mohaddes, K. (2023). Rising Temperatures, Falling Ratings: The Effect of Climate Change on Sovereign Creditworthiness. Management Science, 69(12), 7468–7491.

Ranger, N., Oliver, T., Alvarez, J. et al.(2024). Assessing the materiality of nature-related financial risks for the UK. Green Finance Institute, 135pp.

Ranger, N., Alvarez, J., Freeman, A. et al. (2023). The Green Scorpion: the Macro-Criticality of Nature for Finance. NGFS occasional report

(Remarks on code availability)

Reviewer #3

(Remarks to the Author)
General comments
This manuscript offers an important and innovative contribution by explicitly integrating biodiversity and ecosystem-service loss into sovereign credit risk assessment. The coupling of GTAP–InVEST with a machine-learning replication of S&P's sovereign methodology is novel and compelling, and the headline results, large implied increases in default risk and debt servicing costs, are striking and policy-relevant. The paper is clearly written, methodologically ambitious, and likely to attract significant attention across economics, finance, and environmental policy communities. Thus, I strongly recommend this paper for publication. That said, several issues warrant careful consideration before acceptance.
First, the reliance on three ecosystem services (pollination, fisheries, tropical timber) is well justified technically, but the manuscript would benefit from clearer framing of what this implies substantively. The authors state that impacts are "lower bounds," yet the policy narrative leans heavily on the magnitude of the results. A more explicit discussion of how omitted services (e.g., flood regulation, water supply, soil retention) might change the sign, scale, or regional distribution of effects would help guard against overgeneralization.
Third, the sovereign ratings model itself, while carefully validated, is necessarily an approximation of a proprietary and judgment-heavy process. The random forest predicts ratings largely from GDP per capita and macro-fiscal ratios; biodiversity enters only through its effect on GDP and related aggregates. This raises a conceptual concern: the framework demonstrates that biodiversity loss matters insofar as it reduces GDP and worsens standard fiscal metrics, rather than establishing biodiversity as an independent driver of credit risk. The paper's framing sometimes implies a more direct channel. Clarifying that the contribution is primarily to embed forward-looking environmental shocks into existing macro-fiscal rating logic would improve conceptual precision.
Third, the scenarios themselves are extreme, especially the Partial Ecosystem Collapse case (90% losses across multiple services). As stress tests they are defensible, but the paper occasionally blurs the line between stress-testing and forecasting. Stronger language distinguishing "plausible tail-risk stress tests" from expected trajectories would help prevent misinterpretation by policymakers and the media.
In sum, this is a high-impact and methodologically sophisticated paper that deserves publication after revision. Strengthening the treatment of uncertainty, sharpening the conceptual framing of what is (and is not) being captured in "creditworthiness," and more clearly distinguishing stress-testing from prediction would materially improve the manuscript's rigor and credibility.

Specific points:
Be clearer on why you have 23 countries. And, why not use the other countries that were included as composite regions by just assuming equal impact across the member countries?

Where you write: With at least half of global gross domestic product (GDP) considered moderately or41highly dependent on nature,1 there is mounting evidence that environmental-economic42risks extend well beyond the climate system to include biodiversity loss, deforestation,43water stress, and broader environmental change.2 3
I would make more nuance here. The WEF report on "reliance on nature" is very different from the mechanistic models you cite next, which specify the nature of that reliance.

Where you write "Yet action on biodiversity policy and finance has long lagged climate efforts."
This is a critical and excellent point and so I'm glad to see this so well argued

Where you write "The results in Fig 1 reveal an important feature of the nature-GDP-credit ratings197relationship, which is highly non-linear. This is demonstrated in the rankings of198countries by impacts on GDP versus credit ratings. In terms of impacts on GDP, China199and Malaysia are in the middle of the pack, with shortfalls of 3.1% and 4.2%,200respectively (Fig 1, Panel A). But these translate into the greatest impacts on201sovereign downgrades, with China and Malaysia sliding 5.5 and 6.3 notches,202"
The nonlinearity is a hugely important point. I would expand and clarify this section, possibly with a plot of a "degree of

nonlinearity" derived from the ratio between the scenarios.
Regarding "For investors, we show that forward-looking environmental risks can be incorporated277into mainstream risk measures such as credit ratings, without the need to resort to278poorly defined 'ESG' metrics that often lack scientific underpinnings.279"
This is very important for many practitioners in hear the message that ESG tools are often very flawed. I would suggest emphasizing this more and earlier in the text.
Regarding: "This implementation entails harmonising GTAP–InVEST AEZ-region shocks with362national accounts, propagating nature-adjusted GDP into fiscal/external indicators via363practitioner-based mappings, and embedding the resulting macro paths in a ratings–364probability of default–costs of borrowing pipeline.365
This is the only part of the manuscript where I was confused. The text suggests you are using the "shocks" that went into GTAP-InVEST as inputs to your subsequent calculations? I thought it was the GDP results FROM GTAP-InVEST that were the primary inputs. Please clarify.

(Remarks on code availability)
The Github Repo is very well implemented and organized.

Decision Letter:

1st February 2026

*Please ensure you delete the link to your author homepage in this e-mail if you wish to forward it to your co-authors.

Dear Professor Agarwala,

Your Article, "Biodiversity and the future creditworthiness of nations" has now been seen by three reviewers. You will see from their comments copied below that while they find your work of considerable potential interest, they have raised quite substantial concerns that must be addressed. In light of these comments, we cannot accept the manuscript for publication, but would be very interested in considering a revised version that addresses these concerns.

If you choose to revise your manuscript taking into account all reviewer comments, please highlight all changes in the manuscript text file.

We are committed to providing a fair and constructive peer-review process. Please do not hesitate to contact us if there are specific requests from the reviewers that you believe are technically impossible or unlikely to yield a meaningful outcome.

If revising your manuscript:

* Include a "Response to reviewers" document detailing, point-by-point, how you addressed each referee comment. If no action was taken to address a point, you must provide a compelling argument. This response will be sent back to the referees along with the revised manuscript.

* If you have not done so already we suggest that you begin to revise your manuscript so that it conforms to our Article format instructions at http://www.nature.com/natecolevol/info/final-submission. Refer also to any guidelines provided in this letter.

* Include a revised version of any required reporting checklist. It will be available to referees (and, potentially, statisticians) to aid in their evaluation if the manuscript goes back for peer review. A revised checklist is essential for re-review of the paper.

Please use the link below to submit a revised paper:

Link Redacted

Note: This URL links to your confidential home page and associated information about manuscripts you may have submitted, or that you are reviewing for us. If you wish to forward this email to co-authors, please delete the link to your homepage.

If you wish to submit a suitably revised manuscript we would hope to receive it within 6 months. If you cannot send it within this time, please let us know. We will be happy to consider your revision so long as nothing similar has been accepted for publication at Nature Ecology & Evolution or published elsewhere.

Nature Ecology & Evolution is committed to improving transparency in authorship. As part of our efforts in this direction, we are now requesting that all authors identified as 'corresponding author' on published papers create and link their Open Researcher and Contributor Identifier (ORCID) with their account on the Manuscript Tracking System (MTS), prior to acceptance. This applies to primary research papers only. ORCID helps the scientific community achieve unambiguous attribution of all scholarly contributions. You can create and link your ORCID from the home page of the MTS by clicking on 'Modify my Springer Nature account'. For more information please visit please visit www.springernature.com/orcid.

Please do not hesitate to contact me if you have any questions or would like to discuss the required revisions further.

Thank you for the opportunity to review your work.

Yours sincerely,
[Redacted]

Reviewer expertise:

Reviewer #1: biodiversity economics, ecosystem services

Reviewer #2: fiscal stability, climate risks

Reviewer #3: applied economics, ecosystem services modelling

Reviewer comments:

Reviewer #1 (Remarks to the Author):

Thanks to the authors for this well-written, clear, and important paper.

A few questions/comments, to cut to the chase:
1. The partial ecosystem collapse (PEC): why was a 90% reduction chosen? What rationale for this percentage? I kept wondering what the likelihood of a PEC would be, and what conditions might lead to it. It seems to me that this is crucial information missing from the paper. At present it feels like this 90% is "picked out of a hat" and lacking context. Without a good rationale – linked to some justification - I would even suggest that the PEC scenario should be excluded from the paper.

2.On page 6 the authors point to the non-linear relationship between changes in GDP vs credit ratings. This non-linearity is not explained, nor is the differences between the different countries.

3.Do the authors see any connections between this research and the literature on financial subordination (e.g. https://academic.oup.com/cje/article-abstract/46/4/679/6598707; https://www.tandfonline.com/doi/full/10.1080/09692290.2022.2098359). It seems to me not-insignificant that the countries most impacted are often "subordinated" in the wider global political economy. There might be more insights to draw out here, particularly related to Article 20 of the CBD, which is essentially a common but differentiated responsibility clause, but also connections to development financing questions and the wider efforts to bring into being a multilateral debt mechanism.

4.Is this paper really about biodiversity? As far as I can see, based on the limitations of the INVEST model, this is about ecosystem services, and a very (very) narrow three services. I think it is important to recognize that this is a proxy for biodiversity, but a very imperfect one, particularly for the Nature: Ecology and Evolution audience (and for a paper that self-identifies as being closer to ecology and science than other nature risk work). I would like to see the authors say a bit more about this limited ecological range, particularly as it relates to biodiversity. While the authors suggest that this narrow range of ES modeled means a likely underestimation of impact, I am thinking that a whole lot of biodiversity can be lost before one might see impacts on financial assets

Reviewer #2 (Remarks to the Author):

The paper aims to analyse the impact of biodiversity loss on sovereign creditworthiness using S&P ratings and biodiversity loss impacts derived from GTAP–InVEST.
The assumptions are that sovereign ratings are currently not pricing biodiversity loss in sovereign ratings, and that biodiversity loss is expected to worsen country's economic competitiveness, leading to an increase sovereign debt issuance needed to finance investments in nature restoration. A worsening in sovereign ratings would make it more expensive for countries to borrow on the market, increasing sovereign debt even further.

Genereal comments

The topic is of interest for the finance community as shown by growing research (Garel et al. 2024, Flammer et al. 2025) and a recent special issue on Biodiversity finance (Giglio et al. 2025) published in Review of Finance, and another one published in Ecological Economics (the latter one edited by some of the coauthors of this paper, see Guo et al. 2025). While being of interest, the focus of the paper is not particularly novel, nor is the approach, that includes recent contributions of the authors for the introduction of climate risk into sovereign ratings (Klusak et al. 2023).
In addition, the implementation of the analysis faces several methodological limitations that are not properly discussed nor addressed.

While I am very sympathetic with the authors' view that governments, central banks, financial regulators, and international financial institutions should not ignore nature-related financial risk, I am not persuaded that the approach proposed here would help them in this process.

Specific comments

There are some well known limitations to the inputs the rating model uses to embed biodiversity risk coming from the GTAP–InVEST model, which is a Computable General Equilibrium model (CGE) and are not properly discussed. These include CGE assumptions of linearity of shock, rationale expectations, single optimal intertemporal equilibrium, and lack of finance and finance-economy feedback that are well known in the field and lead to a misestimation of the impact of even large shocks on GDP.

The use of a coupled CGE–nature framework (GTAP–InVEST) to generate economy-wide shocks that propagate through global trade linkages before being fed into the ratings model is not novel and nature VaR estimates already exist at more granular level (see Ranger et al. 2024 for the UK).

The novelty regarding the integration of nature into macro-financial risk analytics should be revised at the light of the growing literature in the field combining ENCORE dependency/exposure mappings with input–output or loan-book data layers and sector–ecosystem service linkages (see NGFS's Green Scorpion, Ranger et al. 2023) and integration of the NVaR into a macroeconomic model with similar features (Nigem, see Ranger et al. 2024).

Scenarios play a key role in financial risk assessment and ratings adjustment. Yet here the scenarios are built exogenously, and there is no real scenario design. Also, it is unclear to what extent if any a main driver of the magnitude of the shock in the economy - i.e. the correlation of ecosystem dimensions and shocks, and their compounding - is modelled and analysed. This is a main limitation as nature and climate risks compound, among themselves and with climate risks (Ranger et al. 2024). Correlation and compound may lead to much larger economic impacts of biodiversity loss results, and through that on the fundamentals used for the financial risk analysis. These limitations are overlooked in the analysis and yet are important to understand the relevance of the shocks in the economy and their transmission to sovereigns.

The long term focus of the analysis via the S&P long-term foreign-currency sovereign ratings may be misleading. Investment decisions have a much shorter time horizon, as finance expectations may adjust abruptly as a result of new information on biodiversity risk exposure of the country, and lead to an adjustment of the credit rating well before the actual biodiversity loss occurs. Thus, long term foreign-currency sovereign ratings may not be the most relevant one.

The results show large impacts, in particular for still high GDP growth countries China and India. I have some concerns regarding the numbers. Ecosystems do not deplete all at once. IPBES (2022) shows that biodiversity degradation is already occurring, and yet the impact on the economy is still unclear. However, we can assume that if main ecosystems resources are missing, in times of growing geopolitical tension and trade barriers that limit import of key raw materials, countries most affected would start to act to build resilience to such losses, to prevent the risk of full collapse. Before a country goes to default, the central bank may also intervene (see the management of the Greek debt crisis last decade). Nevertheless, the analysis does not provide a simulation of the endogenous fiscal policy and monetary policy response that may be well triggered by ecosystem degradation, well before a collapse occurs.

If countries do not do anything, in the case of a partial ecosystem collapse, the related economic impacts would show up in the shorter term. Investors would look at countries' declining fundamentals, and prepare to adjust for that in the rating.An economic downturn would be transmitted to all financial contracts and securities, most likely starting from stocks as a result of worsening firms' performance. Only in case of expectations of a country getting close to default, its sovereign creditworthiness and ratings would massively adjust.
Thus, would a partial ecosystem collapse realise, the role of ratings may be the last ones occurring on the pipeline and the last to care as the economy would have already collapsed.

References

Flammer, C., Giroux, T., & Heal, G. M. (2025). Biodiversity finance. Journal of Financial Economics, 164, 103987.

Garel, A., Romec, A., Sautner, Z., & Wagner, A. F. (2024). Do investors care about biodiversity?. Review of Finance, 28(4), 1151-1186.

Giglio, S., Kuchler, T., Stroebel, J., & Zeng, X. (2025). Biodiversity risk. Review of Finance, rfaf063.

Guo, C., Agarwala, M., FREEMAN, M. C., Groom, B., & McKenzie, E. (2025). Introduction to the special issue:'Biodiversity and finance: Risk, disclosure and double materiality'. Ecological Economics, 240, 108798.

Klusak, P., Agarwala, M., Burke, M., Kraemer, M., & Mohaddes, K. (2023). Rising Temperatures, Falling Ratings: The Effect of Climate Change on Sovereign Creditworthiness. Management Science, 69(12), 7468–7491.

Ranger, N., Oliver, T., Alvarez, J. et al.(2024). Assessing the materiality of nature-related financial risks for the UK. Green Finance Institute, 135pp.

Ranger, N., Alvarez, J., Freeman, A. et al. (2023). The Green Scorpion: the Macro-Criticality of Nature for Finance. NGFS occasional report

Reviewer #3 (Remarks to the Author):

General comments
This manuscript offers an important and innovative contribution by explicitly integrating biodiversity and ecosystem-service loss into sovereign credit risk assessment. The coupling of GTAP–InVEST with a machine-learning replication of S&P's sovereign methodology is novel and compelling, and the headline results, large implied increases in default risk and debt servicing costs, are striking and policy-relevant. The paper is clearly written, methodologically ambitious, and likely to attract significant attention across economics, finance, and environmental policy communities. Thus, I strongly recommend this paper for publication. That said, several issues warrant careful consideration before acceptance.
First, the reliance on three ecosystem services (pollination, fisheries, tropical timber) is well justified technically, but the manuscript would benefit from clearer framing of what this implies substantively. The authors state that impacts are "lower bounds," yet the policy narrative leans heavily on the magnitude of the results. A more explicit discussion of how omitted services (e.g., flood regulation, water supply, soil retention) might change the sign, scale, or regional distribution of effects would help guard against overgeneralization.
Third, the sovereign ratings model itself, while carefully validated, is necessarily an approximation of a proprietary and judgment-heavy process. The random forest predicts ratings largely from GDP per capita and macro-fiscal ratios; biodiversity enters only through its effect on GDP and related aggregates. This raises a conceptual concern: the framework demonstrates that biodiversity loss matters insofar as it reduces GDP and worsens standard fiscal metrics, rather than establishing biodiversity as an independent driver of credit risk. The paper's framing sometimes implies a more direct channel. Clarifying that the contribution is primarily to embed forward-looking environmental shocks into existing macro-fiscal rating logic would improve conceptual precision.
Third, the scenarios themselves are extreme, especially the Partial Ecosystem Collapse case (90% losses across multiple services). As stress tests they are defensible, but the paper occasionally blurs the line between stress-testing and forecasting. Stronger language distinguishing "plausible tail-risk stress tests" from expected trajectories would help prevent misinterpretation by policymakers and the media.
In sum, this is a high-impact and methodologically sophisticated paper that deserves publication after revision. Strengthening the treatment of uncertainty, sharpening the conceptual framing of what is (and is not) being captured in "creditworthiness," and more clearly distinguishing stress-testing from prediction would materially improve the manuscript's rigor and credibility.

Specific points:
Be clearer on why you have 23 countries. And, why not use the other countries that were included as composite regions by just assuming equal impact across the member countries?

Where you write: With at least half of global gross domestic product (GDP) considered moderately or41highly dependent on nature,1 there is mounting evidence that environmental-economic42risks extend well beyond the climate system to include biodiversity loss, deforestation,43water stress, and broader environmental change.2 3
I would make more nuance here. The WEF report on "reliance on nature" is very different from the mechanistic models you cite next, which specify the nature of that reliance.

Where you write "Yet action on biodiversity policy and finance has long lagged climate efforts."
This is a critical and excellent point and so I'm glad to see this so well argued

Where you write "The results in Fig 1 reveal an important feature of the nature-GDP-credit ratings197relationship, which is highly non-linear. This is demonstrated in the rankings of198countries by impacts on GDP versus credit ratings. In terms of impacts on GDP, China199and Malaysia are in the middle of the pack, with shortfalls of 3.1% and 4.2%,200respectively (Fig 1, Panel A). But these translate into the greatest impacts on201sovereign downgrades, with China and Malaysia sliding 5.5 and 6.3 notches,202"
The nonlinearity is a hugely important point. I would expand and clarify this section, possibly with a plot of a "degree of nonlinearity" derived from the ratio between the scenarios.
Regarding "For investors, we show that forward-looking environmental risks can be incorporated277into mainstream risk measures such as credit ratings, without the need to resort to278poorly defined 'ESG' metrics that often lack scientific underpinnings.279"
This is very important for many practitioners in hear the message that ESG tools are often very flawed. I would suggest emphasizing this more and earlier in the text.
Regarding: "This implementation entails harmonising GTAP–InVEST AEZ-region shocks with362national accounts, propagating nature-adjusted GDP into fiscal/external indicators via363practitioner-based mappings, and embedding the resulting macro paths in a ratings–364probability of default–costs of borrowing pipeline.365
This is the only part of the manuscript where I was confused. The text suggests you are using the "shocks" that went into GTAP-InVEST as inputs to your subsequent calculations? I thought it was the GDP results FROM GTAP-InVEST that were the primary inputs. Please clarify.

Reviewer #3 (Remarks on code availability):

The Github Repo is very well implemented and organized.

Version 1:

Reviewer comments:

Reviewer #1

(Remarks to the Author)
I am satisfied with the responses and revisions. Thank you.

(Remarks on code availability)

Reviewer #3

(Remarks to the Author)
The authors have addressed all my points and I believe the article is ready for publication without further changes. Also, reading the other reviewers, they raise very good points about how better to nuance the discussion about "picked from a hat" levels for e.g. the pollination collapse shock. It is definitely true that these are arbitrary, but that is because its nearly impossible to know exactly how a regime change might happen before it happens. Thus, I would argue to that arbitrariness is an acceptable flaw that lets us explore an important scenario space.

(Remarks on code availability)

Decision Letter:

9th March 2026

Dear Dr. Agarwala,

Thank you for submitting your revised manuscript "Biodiversity and the future creditworthiness of nations" (NATECOLEVOL-25124388A). It has now been seen again by two of the original reviewers; their comments are below. The reviewers find that the paper has improved in revision, and therefore we'll be happy in principle to publish it in Nature Ecology & Evolution, pending minor revisions to satisfy the reviewers' final requests and to comply with our editorial and formatting guidelines.

If the current version of your manuscript is in a PDF format, please email us a copy of the file in an editable format (Microsoft Word or LaTex)-- we can not proceed with PDFs at this stage.

If you have not done so already, please ensure that you also email us a completed copy of the Reporting summary :

Reporting summary: https://www.nature.com/documents/nr-reporting-summary.pdf

We are now performing detailed checks on your paper and will send you a checklist detailing our editorial and formatting requirements in about a week. Please do not upload the final materials and make any revisions until you receive this additional information from us.

Thank you again for your interest in Nature Ecology & Evolution. Please do not hesitate to contact me if you have any questions.

Best regards
[Redacted]

Reviewer #1 (Remarks to the Author):

I am satisfied with the responses and revisions. Thank you.

Reviewer #3 (Remarks to the Author):

The authors have addressed all my points and I believe the article is ready for publication without further changes. Also, reading the other reviewers, they raise very good points about how better to nuance the discussion about "picked from a hat" levels for e.g. the pollination collapse shock. It is definitely true that these are arbitrary, but that is because its nearly impossible to know exactly how a regime change might happen before it happens. Thus, I would argue to that arbitrariness is an acceptable flaw that lets us explore an important scenario space.

Open Access This Peer Review File is licensed under a Creative Commons Attribution 4.0 International License, which permits use, sharing, adaptation, distribution and reproduction in any medium or format, as long as you give appropriate credit to the original author(s) and the source, provide a link to the Creative Commons license, and indicate if changes were made.
In cases where reviewers are anonymous, credit should be given to 'Anonymous Referee' and the source.
The images or other third party material in this Peer Review File are included in the article's Creative Commons license, unless indicated otherwise in a credit line to the material. If material is not included in the article's Creative Commons license and your intended use is not permitted by statutory regulation or exceeds the permitted use, you will need to obtain permission directly from the copyright holder.
To view a copy of this license, visit https://creativecommons.org/licenses/by/4.0/

Responses in blue text to Reviewer 1's comments on "Biodiversity and the future creditworthiness of nations"

Many thanks for your helpful comments and suggestions. In what follows we explain how we have addressed your comments in our revised manuscript. For convenience, our response report includes your full comments in black font. Our point-by-point responses appear in bullet points in blue font.

Reviewer #1 (Remarks to the Author):

Thanks to the authors for this well-written, clear, and important paper.

A few questions/comments, to cut to the chase:
1. The partial ecosystem collapse (PEC): why was a 90% reduction chosen? What rationale for this percentage? I kept wondering what the likelihood of a PEC would be, and what conditions might lead to it. It seems to me that this is crucial information missing from the paper. At present it feels like this 90% is "picked out of a hat" and lacking context. Without a good rationale – linked to some justification - I would even suggest that the PEC scenario should be excluded from the paper.

- Thanks for this important comment. We agree that additional justification and explanation of the scenario would be helpful and have made direct changes to the text in addition to the comments to the reviewer here. Our response to the reviewer is structured as follows. First, we clarify that this is a scenario simulation - not a probabilistic prediction - and that it can be thought of as a form of tail risk assessment. Second, we explain the origin of the scenario in the GTAP-InVEST research published by the World Bank[1], and subsequently in PNAS[2]. Third, we point to existing literature to contextualise our 90% (PEC) scenario, and highlight the recent Alaskan snow crab fishery as a real-world example of a 90% collapse in an economically important ecosystem. Finally, we pinpoint specific changes in the text with page and line references.
- **Scenario Justification:** The reviewer raises an important limitation commonly found in scenario analyses: individual scenarios can seem ad hoc, or indeed "picked out of a hat". The PEC scenario is not intended as a forecast or probabilistic statement, but as a tail-risk stress test, analogous to the use of extreme but non-probabilistic scenarios in climate and financial risk analysis[3–6]. Its purpose is to explore system vulnerability and

transmission mechanisms under severe ecosystem service shocks, rather than to predict expected outcomes. This is analogous to the use of the SSP-RCP scenario combinations common to climate research.

Whilst this scenario is grounded in previous research and observable history (see below), we agree that the originally submitted manuscript could leave the reader wondering whether it was in fact ‘picked out of a hat’.

- **Origins in GTAP-InVEST**: The PEC scenario builds directly on the GTAP–InVEST framework developed by the World Bank. As we state in the manuscript (**P4 L18 – 23)**, “*GTAP-InVEST is the first-of-its-kind effort to combine a global, spatially-explicit ecosystem service valuation model (InVEST) with a global computable general equilibrium model (GTAP) to jointly determine how changes in natural capital and ecosystem service provision affect primary industries, scale through value chains, and spread through trade links, to affect macroeconomic outcomes such as GDP*.” In our view (and one regularly expressed at leading conferences and workshops), GTAP-InVEST is among the first and most respected models of its kind. The PNAS paper[2] ranks in the top 5% of research scored by Altmetrics and in the 98th percentile for research of a similar age. Because of the quality and wide reception of this work, we deliberately chose to align our scenarios with theirs to enhance comparability with the seminal literature. Basically, it’s easier for people to contextualise our results if they are based on already familiar (and most importantly - scientifically respected) scenarios.

- **Is 90% (the partial ecosystem collapse) realistic/credible?**: Without making a probabilistic statement, we can point to previous research using similar scenarios, and even a recent real-world example in which a 90% collapse is observed.

  Marine fisheries: The Alaskan snow crab fishery faced a 90% collapse in snow crab populations between 2018-2021. During this time, “*more than 10 billion snow crab have disappeared from the Bering Sea, and the population collapsed to historical lows in 2021*”[7]. This led to a complete closure of the fishery which had previously generated $227m in annual catch, and demonstrates that 90% collapse is not an impossible or even unprecedented experience in marine fisheries.

  Tropical timber: there remains significant uncertainty over the extent, timing, and magnitude of tipping points in tropical timber ecosystems such as the Amazon[8–10]. Lovejoy and Nobre[11,12] note that a tipping point could be imminent. Others suggest it may be over a century away[8].

  Wild Pollination: A 90% collapse in pollinator populations is also an established benchmark used in the scientific literature[13] and indeed studies already document 60% reductions in pollinator abundance in some agricultural ecosystems[14] with some models indicating declines in excess of 90% under worst case scenarios[15].

- As a direct result of your comment, we have added the following text to our paper, **P5, L8-14**:

The approach means our results reflect scenario analyses, not predictions of future ratings. Predictions would require probabilistic statements about the likelihood of each scenario, which are not presently available. The result is therefore analogous to tail-risk stress tests common in environmental economics[31] and practitioner reports[32]. For context, a 90% collapse scenario is consistent with research on pollinators[33–35] and observable history in marine fisheries.[36] There is greater debate over the magnitude and timing of collapse in tropical forests.[37–41]

2.On page 6 the authors point to the non-linear relationship between changes in GDP vs credit ratings. This non-linearity is not explained, nor is the differences between the different countries.

- We appreciate you highlighting this point. The non-linearity we refer to arises directly from the methodologies used by ratings agencies and the structure of sovereign credit ratings. Credit rating agencies assess macroeconomic fundamentals using discrete rating bands or "buckets", including for income and development indicators such as GDP per capita. These bands introduce an inherent non-linearity into the mapping between GDP and ratings: countries located near the centre of a band may experience moderate GDP changes with little effect on their rating, whereas countries positioned near a threshold between bands can experience large rating changes in response to relatively small GDP shocks.
- Our methodology molds around this data generating process and reveals these non-linearities to us. The random-forest model recovers these threshold effects directly from the historical ratings data, revealing a step-like relationship between GDP and credit ratings rather than a smooth linear gradient. This explains why countries with similar GDP impacts can experience very different rating outcomes, and why some countries (e.g. China) appear particularly sensitive to modest GDP losses.
- In response to this comment, we have made direct changes to the text. For convenience, we have pasted the offending paragraph here and underlined the new text (**P7, L3-8**):

"The results in Fig 1 reveal an important feature of the nature-GDP-credit ratings relationship, which is highly non-linear. This is demonstrated in the rankings of countries by impacts on GDP versus credit ratings. <u>Non-linearity in the GDP–ratings relationship arises because ratings are constructed using discrete 'income bands'. Countries near band thresholds can experience large rating changes from modest GDP shocks, whereas countries in the middle of a band show limited sensitivity. Our model recovers these threshold effects directly from the ratings data, explaining the observed cross-country heterogeneity.</u> In terms of impacts on GDP, China and Malaysia are in the middle of the pack, with shortfalls of 3.1% and 4.2%, respectively (Fig 1, Panel A). But these translate into the greatest impacts on sovereign downgrades, with China and Malaysia sliding 5.5 and 6.3 notches, respectively (Fig 1, Panel B). Finally, because these results reflect losses across only a limited range of ecosystem services, the associated impacts should be considered lower-bounds."

- Further discussion of non-linearities is also found in the supplementary material text around figure M3 (**P15, L24 – P16, L9**).

3.Do the authors see any connections between this research and the literature on financial subordination (e.g. https://academic.oup.com/cje/article-abstract/46/4/679/6598707; https://www.tandfonline.com/doi/full/10.1080/09692290.2022.2098359). It seems to me not-insignificant that the countries most impacted are often "subordinated" in the wider global political economy. There might be more insights to draw out here, particularly related to Article 20 of the CBD, which is essentially a common but differentiated responsibility clause, but also connections to development financing questions and the wider efforts to bring into being a multilateral debt mechanism.

- These are important papers on financial subordination. They raise important questions about how global financial structures, development finance, and environmental pressures intersect, particularly for lower-income countries.

  Our analysis is complementary but distinct. We do not make claims about structural bias in sovereign credit ratings, nor do we interpret lower ratings as evidence of financial subordination per se. Instead, our results reflect observable differences in economic structure, fiscal capacity, and exposure to biodiversity-related shocks and how they might propagate through existing rating methodologies. In this sense, the asymmetric impacts we document arise from differential exposure, concentration/diversification, and income levels rather than from an assumed bias in financial markets or rating practices.

  That said, we agree that our findings may intersect with broader political economy debates about development finance and international responsibility, including discussions around common but differentiated responsibilities under Article 20 of the Convention on Biological Diversity. Indeed, it would be exciting if our paper were to be referenced in the field of financial subordination. However, our strong preference is to remain laser focused on our primary objective: demonstrating how environmental science can be incorporated into mainstream financial risk metrics. We demonstrate that this is doable whether or not one adopts the financial subordination framing. We also worry about pushing readers through additional jargon, conceptual frameworks and themes that are not central to the research actually conducted for this paper.

  Moreover, we would be interested in exploring this theme more deliberately in future research, including with some of our applied policy work with the African Union, the African Review Mechanism, and a group of small island states where we currently work. We have some ideas on financial geography that could fit this theme, but would be out of scope for the current manuscript.

  For this paper, our conclusion does however entertain a discussion on the implications and connections with debt markets and development. See **P11, L16 – L23**

“Debt in lower-income, often biodiversity-rich countries that already face among the world’s highest borrowing costs may be especially vulnerable to higher risk premia driven by nature-loss.[20] The UN Secretary General describes the ‘crushing’ debt crisis as “nothing less than a development disaster”.[44] We show that continued nature loss will only exacerbate the challenge, undermining the ability of many biodiversity-rich nations to meet development goals across the board, from education, health, and infrastructure to investments in nature conservation and climate adaptation.“

4.Is this paper really about biodiversity? As far as I can see, based on the limitations of the INVEST model, this is about ecosystem services, and a very (very) narrow three services. I think it is important to recognize that this is a proxy for biodiversity, but a very imperfect one, particularly for the Nature: Ecology and Evolution audience (and for a paper that self-identifies as being closer to ecology and science than other nature risk work). I would like to see the authors say a bit more about this limited ecological range, particularly as it relates to biodiversity. While the authors suggest that this narrow range of ES modeled means a likely underestimation of impact, I am thinking that a whole lot of biodiversity can be lost before one might see impacts on financial assets

- Yes, this is a fair point. And we completely agree: we do not pretend to capture the full richness of biodiversity or the complete array of its potential effects on ratings. Our three ecosystem services would amount to a poor and incomplete proxy for biodiversity at large. As you note, we don’t claim otherwise.

  The limited range of included services reflects current modelling constraints, not a conceptual claim that biodiversity loss is reducible to this extent. These services are among the few for which there is sufficiently mature, spatially explicit, and globally comparable modelling to trace economy-wide impacts through production, trade, and fiscal channels. We think our contribution is thus to present a reproducible model and framework for linking ecosystem service valuation models through to macroeconomically and financially material impacts on creditworthiness of nations.

  Although currently we can only include a very narrow range of biodiversity-dependent ecosystem services, we do touch base with the GTAP In-VEST team at workshops and conferences a few times a year. We know they are working to extend that model to incorporate additional elements of nature. When they do, or indeed as competitor models become available, our replication code stands ready to incorporate more comprehensive results.

  The reviewer is also correct that a lot of biodiversity loss can occur before it filters through to credit ratings and sovereign debt markets. That is why we think our forward-looking assessments, including of extreme scenarios such as the Partial Ecosystem Collapse are important - and important *now.* Financial risk measures that can only tell us something was risky *after* the tipping point has been crossed would be useless. We think it’s a similar difference to getting a diagnosis from a doctor, versus an autopsy report from the coroner.

Financial markets need risk metrics that feel more like a diagnosis, less like an autopsy report.

As a direct response to this comment, we have changed the text in the first paragraph of our discussion (added text is underlined). See **P10, L6-L11**

The continued destruction of natural capital and loss of associated ecosystem services could yield catastrophic financial risks worldwide. We use ecosystem service degradation as a partial and conservative transmission channel linking biodiversity loss to impacts on creditworthiness. While biodiversity encompasses genetic, species, and ecosystem-level dimensions[44], many of which may decline without immediate economic effects, our results suggest that declines in just these three (tropical timber, marine fisheries, and wild pollination) services could be financially material. As natural capital continues to decline,[9] credit ratings that ignore scientific information about nature-related risks may obscure financially material vulnerabilities. We calculate the impacts of losses in marine fisheries catch, tropical timber, and wild pollination services on sovereign credit ratings, the cost of public borrowing, and the probability of sovereign default under a business-as-usual and partial collapse scenario.

Finally, we also acknowledge that in a 90% collapse scenario, we'd expect humanity to have bigger concerns than a sovereign downgrade. We wouldn't want to present credit ratings as the primary reason for preventing biome collapse (see humorous figure). Rather, we're trying to demonstrate that nature-loss is financially material, and that even with existing (incomplete) knowledge, we can nevertheless construct science-based financial risk measures that are familiar and interpretable to financial markets.

[redacted]

Ultimately, if we accept that nature loss is bad for the economy, if we think that link is worth understanding and communicating to financial markets, and if we think that understanding and communication should be grounded in environmental science and economics, then we hope this paper makes a positive (if incomplete) contribution.

Responses in blue text to Reviewer 2's comments on "Biodiversity and the future creditworthiness of nations"

Many thanks for your helpful comments and suggestions. In what follows we explain how we have addressed your comments in our revised manuscript. For convenience, our response report includes your full comments in black font. Our point-by-point responses appear in bullet points in blue font.

Reviewer #2 (Remarks to the Author):

The paper aims to analyse the impact of biodiversity loss on sovereign creditworthiness using S&P ratings and biodiversity loss impacts derived from GTAP–InVEST.
The assumptions are that sovereign ratings are currently not pricing biodiversity loss in sovereign ratings, and that biodiversity loss is expected to worsen country's economic competitiveness, leading to an increase sovereign debt issuance needed to finance investments in nature restoration. A worsening in sovereign ratings would make it more expensive for countries to borrow on the market, increasing sovereign debt even further.

- This is a fair summary of the paper and we appreciate your insightful comments. Two minor clarifications are:
    - We do not assume biodiversity loss reduces economic *competitiveness*, rather we incorporate a GTAP-InVEST result that biodiversity loss would reduce economic *output*. We are very intentional with our use of that word.
    - Our model makes no assumptions about future debt issuance. Our results show that biodiversity loss would increase the cost of future debt, but we do not attempt to predict (a) future levels of debt issuance or (b) how future debt will be deployed (e.g. towards investments in nature restoration versus other purposes). The reviewer is correct that our discussion mentions potential responses (specifically, the final four paragraphs of Section 3), but this is discussion rather than an assumption.

Genereal comments

The topic is of interest for the finance community as shown by growing research (Garel et al. 2024, Flammer et al. 2025) and a recent special issue on Biodiversity finance (Giglio et al. 2025) published in Review of Finance, and another one published in Ecological

Economics (the latter one edited by some of the coauthors of this paper, see Guo et al. 2025). While being of interest, the focus of the paper is not particularly novel, nor is the approach, that includes recent contributions of the authors for the introduction of climate risk into sovereign ratings (Klusak et al. 2023).
In addition, the implementation of the analysis faces several methodological limitations that are not properly discussed nor addressed.
While I am very sympathetic with the authors' view that governments, central banks, financial regulators, and international financial institutions should not ignore nature-related financial risk, I am not persuaded that the approach proposed here would help them in this process.

- Thank you for flagging the general rising interest in these topics, including in top finance journals. We agree this demonstrates a growing awareness of the macro-financial importance of nature-related risks, and believe this reinforces the relevance of our work.
- The reviewer is correct to flag similarities with Klusak et al (2023)[16]. However, we argue the approach in this paper is different for several key reasons:
    1. **Tail risk vs chronic change**: The scenarios modelled in this paper are fundamentally different to those in Klusak et al (2023). The PEC scenario is a tail-risk scenario. Applying the model in this context demonstrates its ability to function as a stress-test. In contrast, Klusak et al., (2023) modelled chronic changes in climate over the century under a range of warming scenarios such as RCP2.6.
    2. **Country coverage**: the countries under study and the extent of their losses enable us to show just how important ratings are in this context. Whilst for some countries, income losses from biodiversity loss may seem modest, their impacts on ratings are disproportionately large (e.g. China). Applying the ratings model to this study, and set of scenarios, provides essential insights into how income losses may map onto rating outcomes for different countries, who each have different starting points on the ratings scale.
    3. **Market-relevant analytics**: we provide additional extensions to the way in which Klusak et al (2023) translate rating outcomes into debt costs and default probabilities. Klusak et al (2023) imposed a constant relationship between ratings and yield spreads. Each one-notch downgrade mechanically translated into a 0.08 - 0.12% increase in yield spreads based on Afonso et al., (2012)[17] and Gande and Parsley (2005)[18]. This relationship was imposed regardless of where the rating was on the scale initially: i.e. they assumed the same impact on yield spreads for a move from AAA to AA+ as for a move from BBB- (the lowest investment-grade) to BB+ (the highest speculative grade). By contrast, in the present paper we employ market option-adjusted spreads data and data on sovereign defaults from S&P to provide more accurate and realistic measures of the severity of certain rating changes for certain countries.
    4. **Default probabilities**: Klusak et al (2023) report results for climate impacts on sovereign ratings and cost of borrowing, but do not provide results for probability of default. Here, we do.

5. **Biodiversity loss versus climate change**: Beyond the modelling and conceptual distinctions outlined above, the most obvious difference is that whilst Klusak et al (2023) deals exclusively with climate risk, here we deal with biodiversity-related risk. As many of the references supplied by the reviewer point out, biodiversity-related financial risks remain badly under-researched in their own right, and in comparison to climate risks (Karolyi and Tobin-de la Puente 2023; Ranger et al 2023; Ranger et al 2024)[4,19,20].

- With respect to helping practitioners: we accept it can be difficult to understand how key stakeholders/end-users can connect with and use scientific research. We believe our research is highly relevant for the asset management, investment, and broader practitioner and regulatory community. Investors are increasingly concerned about tail risks. They can estimate familiar macroeconomic risks pretty well themselves, because they have a deep pool of experience on which to draw. This is not the case for new risks, ranging from unprecedented geopolitical changes to environmental degradation. Our research provides order of magnitude estimates and demonstrates convincingly that a loss of biodiversity services is anything but a trivial risk.

  For example, while ESG and related issues have lost some political traction since their peak some five years ago, Morningstar estimates that global sustainable mutual fund and ETF assets were still around 3.5-3.7 trillion dollars in 2025[21]. Not a trivial amount.

- We also have a degree of experience, including engaging with credit ratings agencies (one of our co-authors served as S&P Global's Chief Sovereign Ratings Officer), central banks, national governments (e.g. the Biden-Harris Administration referenced preliminary/working paper versions of this research in drafting and implementing national policy; at the request of the Biden-Harris Administration, one of our co-authors joined Yale's Tobin Center for Economic Policy to contribute to US policy in this field), and multilateral development banks (e.g. the Asian Infrastructure Investment Bank included a section on nature and sovereign debt based on preliminary/working paper versions of this paper as a chapter in its 2023 flagship report).

  Of course, we do not wish to overclaim, but our experience suggests the methods and approaches contained in this research can in some cases be helpful to key stakeholders.

Specific comments

There are some well known limitations to the inputs the rating model uses to embed biodiversity risk coming from the GTAP–InVEST model, which is a Computable General Equilibrium model (CGE) and are not properly discussed. These include CGE assumptions of linearity of shock, rationale expectations, single optimal intertemporal

equilibrium, and lack of finance and finance-economy feedback that are well known in the field and lead to a misestimation of the impact of even large shocks on GDP.

- We agree with the Reviewer that (i) there are important well-known limitations of CGE models and (ii) that we did not sign-post these sufficiently in the original manuscript.
- As a direct result of this comment, we have changed the manuscript by adding the following paragraph to the Methods Section 6 (**P13, L26 – P14, L3)**.

Whilst GTAP-InVEST constitutes a step change for integrated ecological-economic modelling, a range of assumptions and limitations remain. These arise from the nature of CGE modelling in general, the specific GTAP CGE model itself, the InVEST model, and issues encountered when linking models across multiple spatial scales. First, in its current form, GTAP-InVEST can only be used for comparative static analyses: it can compare a baseline equilibrium against future equilibria, but is not a dynamic model that can endogenise environmental-economic-fiscal feedback loops over time. Second, the three ecosystem services included here are only a limited sub-set of the full spectrum of economically valuable services generated by nature. Third, and relatedly, many of the benefits generated by nature accrue to humans outside of formal markets, and would not be captured in this analysis. Fourth, at present the GTAP-InVEST model does not include a coupled, integrated financial sector. Finally, CGE models entail a range of assumptions for key parameter values, including total factor productivity growth, substitution elasticities, representative household behaviour (in this case Cobb-Douglas utility functions), and production frameworks (in this case, nested constant elasticities of substitution production functions). Accordingly, we believe our results should be considered lower-bound estimates and interpreted as scenario simulations rather than predictions.

The use of a coupled CGE–nature framework (GTAP–InVEST) to generate economy-wide shocks that propagate through global trade linkages before being fed into the ratings model is not novel and nature VaR estimates already exist at more granular level (see Ranger et al. 2024 for the UK).

- After reflecting on this among the authoring team, we respectfully disagree with the Reviewer on this point. First, on the issue of novelty, we are not aware of any other research that connects GTAP-InVEST to sovereign credit ratings simulation. We are familiar with the excellent work by Ranger and colleagues - indeed, one of our co-authors is acknowledged in that report for providing peer-review. For clarification, the Ranger et al., (2024) reference does not:
    - employ GTAP-InVEST
    - mention sovereign credit ratings
    - consider any country beyond the UK.

  We do all of these things.

  Whilst we accept that we are not the first to employ GTAP-InVEST, we believe we *are* the first to employ it to assess how biodiversity loss could affect future sovereign creditworthiness.

Also, Ranger et al (2024) covers the UK, whereas we produce results for 23 countries. For Ranger et al, GDP impacts are the final *output.* For us, GDP impacts are an *input* from which we develop sovereign debt market metrics as an output. Their nature VaR estimates focus on seven UK *commercial* banks, we report results on *sovereign debt and ratings*.

For these reasons, we do not agree that Ranger et al., (2024) - exciting as that work is - in any way reduces the novelty of our manuscript.

The novelty regarding the integration of nature into macro-financial risk analytics should be revised at the light of the growing literature in the field combining ENCORE dependency/exposure mappings with input–output or loan-book data layers and sector–ecosystem service linkages (see NGFS's Green Scorpion, Ranger et al. 2023) and integration of the NVaR into a macroeconomic model with similar features (Nigem, see Ranger et al. 2024).

- Thank you for these suggestions; the reviewer is right to point out these omissions in our review. We have referenced both of these works.

Scenarios play a key role in financial risk assessment and ratings adjustment. Yet here the scenarios are built exogenously, and there is no real scenario design. Also, it is unclear to what extent if any a main driver of the magnitude of the shock in the economy - i.e. the correlation of ecosystem dimensions and shocks, and their compounding - is modelled and analysed. This is a main limitation as nature and climate risks compound, among themselves and with climate risks (Ranger et al. 2024). Correlation and compound may lead to much larger economic impacts of biodiversity loss results, and through that on the fundamentals used for the financial risk analysis. These limitations are overlooked in the analysis and yet are important to understand the relevance of the shocks in the economy and their transmission to sovereigns.

- This is another insightful comment. The reviewer is correct that we have not included the potential compounding effects of climate change & nature loss, and that these correlations matter. The peer-reviewed research literature on this topic is still in its infancy. Perhaps the most important are Bastien-Olvera and Moore[22–25] who created the GREEN-DICE model. Green-DICE explicitly integrates natural capital into the DICE IAM, and demonstrates - as the Reviewer suggests - that the compounding effects of climate change and natural capital loss are important. Unfortunately for the current study, this work does not yet link to specific ecosystems, values, and locations. As such, we elected to go with GTAP-InVEST. As Bastien-Olvera and Moore style literature develops, we'd like to include integrated climate & nature scenarios.

For the present paper, we would expect compounding to increase the magnitude of economic losses, therefore increasing the magnitude of the effect on ratings (ie leading to even bigger downgrades). As such, our results should be considered conservative estimates.

- As a direct consequence of this comment, we have added text to the discussion to address this (new text underlined). **P12, L10 - 13**

A key area for urgent collaboration is in the development of models that link environmental change to ecosystem service provision to economic outcomes. The GTAP-InVEST model on which our results are based is an important example. However, three key gaps remain. First, GTAP-InVEST facilitates the scenario analyses presented here, but does not support probabilistic statements about the likelihood of any given scenario coming to fruition. Thus, our results should be considered simulations rather than predictions. Second, whilst we have focused on biodiversity here, greater effort is needed to model and assess the combined, and potentially compounding effects of climate change and nature loss simultaneously. The Green-DICE model represents an important step, but does not yet provide global results at the same resolution as GTAP-InVEST.[50–53] Third, more work is needed to evidence the environmental benefits of conservation interventions at a macro-scale so that these can be incorporated into financial risk assessments. Currently, countries using debt finance to support conservation and adaptation investments, may face downward pressure on credit ratings, as debt-to-GDP ratios increase and scarce resources are deployed in pursuit of nature-enhancing investments for public goods rather than traditionally productivity-enhancing investments for market returns. An important next step would be to combine GTAP-InVEST-type models with unified 'pressures-and-conservation-impact' (PACI) models[54] that can incorporate the *net* effect of environmental change, accounting for both the negative consequences of nature-loss and the positive benefits from conservation spending.

The long term focus of the analysis via the S&P long-term foreign-currency sovereign ratings may be misleading. Investment decisions have a much shorter time horizon, as finance expectations may adjust abruptly as a result of new information on biodiversity risk exposure of the country, and lead to an adjustment of the credit rating well before the actual biodiversity loss occurs. Thus, long term foreign-currency sovereign ratings may not be the most relevant one.

- The issue of timing and information absorption is apt. There are a couple of things going on. 1) yes, investment decisions on any asset class can be made on short time horizons, especially in liquid markets. So, if the biodiversity risk exposure of a country was revealed in new information, many *market actors* could adjust *before* the biodiversity loss occurs. This efficient market hypothesis argument would entail financial markets adjusting instantaneously (or at the very least, very quickly), with perfect (or at the very least, better and new) information.

But the fact that *many market actors could* adjust prior to biodiversity loss does not mean that *sovereign credit ratings would*. Real-world experience, evidence, and pure practicalities suggest this is unlikely. For instance, consider the COVID-19 pandemic - a major global economic shock with significant fiscal and creditworthiness implications (Tran et al., 2021)[21]. News of the virus first hit international headlines in Jan 2020. By October 2020, the IMF forecast a global economic contraction of 4.4% for that year (this compares with a 0.1% global contraction in the Great Financial Crisis, GFC). Yet, in the 6-14 months following the outbreak there had been fewer downgrades (as a percentage of rated sovereigns) than were observed in the GFC (Tran et al., 2021). There are several potential reasons for this: ratings agencies could have become more cautious following the GFC, not wanting to exacerbate market turmoil by issuing widespread downgrades; frequencies of ratings reviews are subject to regulation, with dates set a year ahead – agencies may have preferred a business-as-usual schedule for ratings announcements; ratings agencies may have been reluctant to act without more information; or they may have thought the pandemic did not affect creditworthiness. What matters here is that we have clear evidence that just because market actors act, it does not follow that credit ratings also change.

Indeed, Ranger et al., (2023, p5) clearly state that. "*Today, nature risks are not priced into financial markets and are not accounted for in the scenarios used by financial institutions, Central Banks and supervisors to date, leaving the financial system exposed to potential systemic risks, as well as contributing to the misalignment of capital flows with societal goals*."

Returning to biodiversity loss, let's think through what would need to happen for the Reviewer's process to unfold. That is, what's the mechanism whereby weight observe "an adjustment of the credit rating well before the actual biodiversity loss occurs"? The steps would need to include, at a minimum: (i) that ratings agencies know such risks exist, (ii) they'd need a credible measure of the economic magnitude of such a risk, (iii) they'd need a sovereign ratings method that is capable of incorporating those magnitudes into the assessment, (iv) they'd need to release that method publicly to markets and regulators (a requirement by law), (v) they'd need to train analysts on these risks, and (vi) they'd need to issue new biodiversity-adjusted ratings.

However, beyond publicly acknowledging that nature-related risks may be material, none of the rest of these criteria are met. S&P Global's sovereign rating method (S&P 2017)[26] does not include biodiversity or nature-related risk. We know that biodiversity loss is occurring at pace, and all around the world, but that sovereign credit ratings methods have not incorporated the associated risk. do not share the position that ratings already factor-in biodiversity-related risk.

Of course, in other comments the Reviewer correctly points to market responses by investors. Garel et al., (2024)[27] provide evidence that public equity (rather than sovereign

bond) investors may care about biodiversity, possibly for up to 72 hours after an information shock. However, this is not the transmission mechanism we study in our paper. We instead focus on the loss of productive ecosystem services that support macroeconomic fundamentals. That is, we examine not an investor response to news headlines, but a real-economy response to changes in the biosphere's capacity to support environmental-economic production.

- We argue that the long term foreign-currency rating is the appropriate measure for assessing the effect of nature-loss on sovereign credit ratings, default probabilities, and the cost of borrowing for a few key reasons.
  - **Headline indicator**: the long-term foreign currency rating is the headline measure that matters the most for investors. It is what is meant by conventional reference to 'sovereign ratings'. This is also the rating that is embedded into regulation, stress tests, and even pension funds' charters governing the quality of debt that can be held as collateral under the Basel Framework.
  - **It is fundamental to sovereign governments**: there is no escape for sovereigns - defaulting on a currency they control is different to one they don't.
  - **Long-termism is important**: excessive short-termism in financial markets is a key driver of multiple market failures, including climate change, biodiversity loss, and chronic underinvestment in infrastructure and a key theme in the field of environmental finance. A good synopsis is the now famous speech by then Governor of the Bank of England (now Prime Minister of Canada) Mark Carney called The Tragedy of the Horizon.

    More directly related to our study is the fact that sovereigns are increasingly offering longer-term debt products. We now even see century bonds in a number of countries. However, despite growing maturities, we don't actually observe new ultra-long term ratings products offered by ratings agencies.

The results show large impacts, in particular for still high GDP growth countries China and India. I have some concerns regarding the numbers. Ecosystems do not deplete all at once. IPBES (2022) shows that biodiversity degradation is already occurring, and yet the impact on the economy is still unclear.

- It is certainly true that China and India would be considered high-growth economies. It is also true that not all ecosystems "deplete all at once". Despite this, ecological tipping points remain a major tail risk concern and there are numerous examples of ecosystems depleting rapidly. Indeed, Ranger et al., (2024) flag tipping points in tropical timber, fisheries, and pollinators as potentially material risks.

  The scenarios considered in our study are well within the realm of possibility and are entirely consistent with those explored in other world-leading research.

  The Partial Ecosystem Collapse (PEC) scenario builds directly on the GTAP–InVEST

framework developed by the World Bank & Justin Johnson's team. As we state in the manuscript (**P4, L18 – L23)**, "*GTAP-InVEST is the first-of-its-kind effort to combine a global, spatially-explicit ecosystem service valuation model (InVEST) with a global computable general equilibrium model (GTAP) to jointly determine how changes in natural capital and ecosystem service provision affect primary industries, scale through value chains, and spread through trade links, to affect macroeconomic outcomes such as GDP*." In our view (and one regularly expressed at leading conferences and workshops), GTAP-InVEST is among the first and most respected models of its kind. The PNAS paper ranks in the top 5% of research scored by Altmetrics and in the 98th percentile for research of a similar age. Because of the quality and wide reception of this work, we deliberately chose to align our scenarios with theirs to enhance comparability with the seminal literature. Basically, it's easier for people to contextualise our results if they are based on already familiar (and most importantly - scientifically respected) scenarios.

- **Is 90% (the partial ecosystem collapse) realistic/credible?**: Without making a probabilistic statement, we can point to previous research using similar scenarios, and even a recent real-world example in which a 90% collapse is observed.

  Marine fisheries: The Alaskan snow crab fishery faced a 90% collapse in snow crab populations between 2018-2021. During this time, "*more than 10 billion snow crab have disappeared from the Bering Sea, and the population collapsed to historical lows in 2021*"[7]. This led to a complete closure of the fishery which had previously generated $227m in annual catch, and demonstrates that 90% collapse is not an impossible or even unprecedented experience in marine fisheries.

  Tropical timber: there remains significant uncertainty over the extent, timing, and magnitude of tipping points in tropical timber ecosystems such as the Amazon[8–10]. Lovejoy and Nobre[11,12] note that a tipping point could be imminent. Others suggest it may be over a century away[8].

  Wild Pollination: A 90% collapse in pollinator populations is also an established benchmark used in the scientific literature[13] and indeed studies already document 60% reductions in pollinator abundance in some agricultural ecosystems[14] with some models indicating declines in excess of 90% under worst case scenarios[15].

- As a direct result of your comment, we have added the following text to our paper – see **P5, L8 - 14**:

The approach means our results reflect scenario analyses, not predictions of future ratings. Predictions would require probabilistic statements about the likelihood of each scenario, which are not presently available. The result is therefore analogous to tail-risk stress tests common in environmental economics[31] and practitioner reports[32]. For context, a 90% collapse scenario is consistent with research on pollinators[33–35] and observable history in marine fisheries.[36] There is greater debate over the magnitude and timing of collapse in tropical forests.[37–41]

However, we can assume that if main ecosystems resources are missing, in times of growing geopolitical tension and trade barriers that limit import of key raw materials, countries most affected would start to act to build resilience to such losses, to prevent the risk of full collapse.

- We think this is one of the central points of our paper - to demonstrate that if ecosystems collapse, there will be negative macroeconomic consequences for economies. Therefore, countries should start to act now to mitigate the likelihood of such a collapse and to build resilience to such losses that do occur.
- However, we do not share the Reviewer's position that we can assume countries will act to prevent collapse. Observable history shows that ecosystems can and do collapse even when policymakers are forewarned.

Before a country goes to default, the central bank may also intervene (see the management of the Greek debt crisis last decade). Nevertheless, the analysis does not provide a simulation of the endogenous fiscal policy and monetary policy response that may be well triggered by ecosystem degradation, well before a collapse occurs.

- We appreciate this point. The Reviewer is correct that we do not conduct a full assessment of potential ex-ante fiscal and monetary policy responses available to countries in advance of a partial collapse. Our research question is different. We explore what the effects of various biodiversity scenarios would be on sovereign credit ratings, not 'what fiscal and monetary policy strategies should countries pursue to prevent ecosystem collapse?'. We agree this would also be a fantastic research question, but is outside the scope of this paper, which is about biodiversity loss and credit ratings.
- In effect, we are conducting tail-risk scenario analyses. We acknowledge the Reviewer's point that policy could eliminate environmental-economic tail risks. Indeed, we'd very much like to see policy do just that.

If countries do not do anything, in the case of a partial ecosystem collapse, the related economic impacts would show up in the shorter term. Investors would look at countries' declining fundamentals, and prepare to adjust for that in the rating.An economic downturn would be transmitted to all financial contracts and securities, most likely starting from stocks as a result of worsening firms' performance. Only in case of expectations of a country getting close to default, its sovereign creditworthiness and ratings would massively adjust.
Thus, would a partial ecosystem collapse realise, the role of ratings may be the last ones occurring on the pipeline and the last to care as the economy would have already collapsed.

- Agreed. Ratings would indeed seem like a small concern in such a scenario, as they ought to be. The value in this analysis is not to point out that ratings will be bad for countries whose economies have collapsed - the value is to point out that some economies are more vulnerable to nature-related economic losses than may appear to be the case based purely on the headline GDP losses. Whilst we agree with your concern, it could easily be translated onto all climate- and nature-related risk assessments: if life on earth ends, economic outcomes might not be important.

- We also note that a previous comment suggested ratings would adjust so fast that the **long-term** rating is an inappropriate metric, but that this comment suggests ratings "may be the last ones occurring.." and that "the economy would already have collapsed".

References added

Flammer, C., Giroux, T., & Heal, G. M. (2025). Biodiversity finance. Journal of Financial Economics, 164, 103987.

Garel, A., Romec, A., Sautner, Z., & Wagner, A. F. (2024). Do investors care about biodiversity?. Review of Finance, 28(4), 1151-1186.

Giglio, S., Kuchler, T., Stroebel, J., & Zeng, X. (2025). Biodiversity risk. Review of Finance, rfaf063.

Guo, C., Agarwala, M., FREEMAN, M. C., Groom, B., & McKenzie, E. (2025). Introduction to the special issue:'Biodiversity and finance: Risk, disclosure and double materiality'. Ecological Economics, 240, 108798.

Klusak, P., Agarwala, M., Burke, M., Kraemer, M., & Mohaddes, K. (2023). Rising Temperatures, Falling Ratings: The Effect of Climate Change on Sovereign Creditworthiness. Management Science, 69(12), 7468–7491.

Ranger, N., Oliver, T., Alvarez, J. et al.(2024). Assessing the materiality of nature-related financial risks for the UK. Green Finance Institute, 135pp.

Ranger, N., Alvarez, J., Freeman, A. et al. (2023). The Green Scorpion: the Macro-Criticality of Nature for Finance. NGFS occasional report

Responses in blue text to Reviewer 3's comments on "Biodiversity and the future creditworthiness of nations"

Many thanks for your helpful comments and suggestions. In what follows we explain how we have addressed your comments in our revised manuscript. For convenience, our response report includes your full comments in black font. Our point-by-point responses appear in bullet points in blue font.

Reviewer #3 (Remarks to the Author):

General comments

This manuscript offers an important and innovative contribution by explicitly integrating biodiversity and ecosystem-service loss into sovereign credit risk assessment. The coupling of GTAP–InVEST with a machine-learning replication of S&P's sovereign methodology is novel and compelling, and the headline results, large implied increases in default risk and debt servicing costs, are striking and policy-relevant. The paper is clearly written, methodologically ambitious, and likely to attract significant attention across economics, finance, and environmental policy communities. Thus, I strongly recommend this paper for publication. That said, several issues warrant careful consideration before acceptance.

- We are extremely grateful to the Reviewer for this constructive and thoughtful review. We are delighted that you also see novelty and practical relevance to key stakeholders. And we are grateful for the chance to make the improvements you suggest.

First, the reliance on three ecosystem services (pollination, fisheries, tropical timber) is well justified technically, but the manuscript would benefit from clearer framing of what this implies substantively. The authors state that impacts are "lower bounds," yet the policy narrative leans heavily on the magnitude of the results. A more explicit discussion of how omitted services (e.g., flood regulation, water supply, soil retention) might change the sign, scale, or regional distribution of effects would help guard against overgeneralization.

- Thanks - this is a fantastic point, but also a devilishly difficult comment to address. We could hazard a guess at how including water supply/quality, soil, flood, air quality, etc might affect the results. We think it's unlikely that the sign would change, but the scale and regional distribution of effects almost certainly would. Our weakness is that we just don't know. The relationships between biodiversity loss and ecosystem service provision, between ecosystem service provision and macroeconomic outcomes (including through global trade), and between GDP impacts and credit ratings are so complex and so non-linear that we're simply not comfortable speculating. We'd need to see these services

modeled out properly before we could responsibly go on record with a claim. However, we do think this is important and as a direct consequence of this comment have changed the text in the main manuscript (page 4, whole paragraph pasted here for convenience, new text underlined): **P4, L24 - 36**

Under a range of scenarios, GTAP-InVEST estimates GDP losses associated with declines in the provision of multiple ecosystem services: marine fisheries, wild pollination services, and tropical timber production. As we explain in greater detail in Section 6 "Methods," the choice of these ecosystems services is predetermined by their ability to be modelled endogenously as sectoral productivity shocks that meet two conditions: (i) the availability of globally consistent, spatially explicit data; and (ii) a clear functional link between biophysical change and sectoral economic productivity. Although other InVEST modules exist, such as water yield, flood regulation, or soil retention, the lack of harmonised global data and the difficulty of mapping them consistently to market sectors prevent their integration into the coupled CGE model. <u>We would expect the inclusion of additional components of biodiversity and ecosystem services to affect the magnitude and relative geographic distribution of our results, although these can be devilishly difficult to model.</u>

Third, the sovereign ratings model itself, while carefully validated, is necessarily an approximation of a proprietary and judgment-heavy process. The random forest predicts ratings largely from GDP per capita and macro-fiscal ratios; biodiversity enters only through its effect on GDP and related aggregates. This raises a conceptual concern: the framework demonstrates that biodiversity loss matters insofar as it reduces GDP and worsens standard fiscal metrics, rather than establishing biodiversity as an independent driver of credit risk. The paper's framing sometimes implies a more direct channel. Clarifying that the contribution is primarily to embed forward-looking environmental shocks into existing macro-fiscal rating logic would improve conceptual precision.

- Yes, this is correct. S&P's rating method (written by one of the co-authors of this manuscript) does contain both 'hard' (quantitative) and 'soft' (more subjective and qualitative) information. It is not a purely mathematical formula that can necessarily be replicated by any random user. Analysts and expertise matter.
- When we embarked on this work, we set ourselves three rules of engagement. First, we would not skimp on the ecological science - that needs to be visible and traceable throughout. Second, we would not 'break the rules' of statistics and econometrics - that is, we won't 'overfit' the model just to gain predictive accuracy. And finally, we wanted to remain as close as possible to the way ratings agencies actually think about sovereign risk and construct sovereign ratings. This final point is important to us, and to potential readers, because it ensures the results are as 'familiar' and 'digestable' as possible. Regulators, governments, investors, pension funds - all know what to do with a sovereign rating (or changes in it).

  We did not want to create an entirely new metric, and then play the game of trying to sell it or convince others to use 'our method'. We do not assume that we are better at sovereign

credit assessment than the professionals and world-leading firms who do this for a living. Rather, we think we can augment their work by producing a method to incorporate a (very slightly) more comprehensive suite of risks.

That said, we absolutely have thought about how we would re-design ratings if we started from scratch. In such an approach, we would certainly explore doing as the Reviewer suggests and identify climate change and nature loss as their own source of risk – a 6th pillar to the S&P model - rather than restricting them only to operate through impacts on GDP. In short, we agree that there's something more to be done here, but we don't think it fits in this (much narrower) paper.

Third, the scenarios themselves are extreme, especially the Partial Ecosystem Collapse case (90% losses across multiple services). As stress tests they are defensible, but the paper occasionally blurs the line between stress-testing and forecasting. Stronger language distinguishing "plausible tail-risk stress tests" from expected trajectories would help prevent misinterpretation by policymakers and the media.

- Thank you for raising this extremely important point. You are correct: we do not predict or forecast ratings and did not intend to imply that we do. With permission, we would like to adopt the terminology you suggest, specifically "plausible tail-risk stress tests" to help prevent misinterpretation (and to doubly ensure we do not overclaim).

  As a direct consequence of this comment, we have changed the text (And used your phraseology, if that's ok?). The following new text appears, end of Section 2, **P5, L8 - 14**:

The approach means our results reflect scenario analyses, not predictions of future ratings. Predictions would require probabilistic statements about the likelihood of each scenario, which are not presently available. The PEC scenario serves as a plausible tail-risk stress test, as is common in environmental economics[31] and practitioner reports[32,33]. For context, a 90% collapse scenario is consistent with research on pollinators[34–36] and observable history in marine fisheries.[37] There is greater debate over the magnitude and timing of collapse in tropical forests.[38–42]

In sum, this is a high-impact and methodologically sophisticated paper that deserves publication after revision. Strengthening the treatment of uncertainty, sharpening the conceptual framing of what is (and is not) being captured in "creditworthiness," and more clearly distinguishing stress-testing from prediction would materially improve the manuscript's rigor and credibility.

- Thank you. Your comments have been intellectually stimulating and greatly improved the manuscript.

Specific points:
Be clearer on why you have 23 countries. And, why not use the other countries that were included as composite regions by just assuming equal impact across the member countries?

- Yea, we agonised over this. You're right. We might be able to claim to cover more countries by using aggregated regions and then disaggregate across countries. I'm sure we'd get more citations and have more of a 'blockbuster' paper. But it would force us into some difficult compromises and possibly ad-hoc assumptions. For instance, what criteria should we use to distribute impacts across countries in the region? We could assign to each country in the region, a share of the region's losses proportional to the country's:
    - **GDP** - surely this is the first that comes to mind. But is it really appropriate? Could we really claim that fisheries collapse would hit Bermuda (per capita GDP $139k) 20 times harder than Jamaica (per capita GDP $7k) just because Bermuda is rich and Jamaica poor? In fact, maybe it goes the other way? Maybe Jamaica is more dependent on fisheries than Bermuda and therefore more exposed (so now we're not even talking about GDP anymore, but exposure of GDP to specific risks)?
    - **Population**: also a good candidate, but ratings don't seem to show any correlation with population (though I suspect the loss of ecosystem service value might?). We know climate damages scale with population - the more people you have, the more people suffer from climate. Ecosystem services might have a similar trend, but China won't lose more coral reef just because they are populous.
    - **Geographic size:** no, *this* one has to be the most important because it scales most directly with ecosystem service provision. But is the US more at risk just because it's larger than Cuba?

- Ultimately, we don't have a very convincing rationale for choosing between these. And we would inevitably lose much of the country-level tailor-made analysis that is possible because of GTAP-InVEST if we started applying these clunky distributional rules.

  In the end, we elected to pursue a smaller set of countries but with greater precision and clarity. We are extremely optimistic that the GTAP-InVEST team will keep expanding that model's abilities and so we could always update later (and as you note, the code is available for others to play along).

Where you write: With at least half of global gross domestic product (GDP) considered moderately or41highly dependent on nature,1 there is mounting evidence that environmental-economic42risks extend well beyond the climate system to include biodiversity loss, deforestation,43water stress, and broader environmental change.2 3
I would make more nuance here. The WEF report on "reliance on nature" is very different from the mechanistic models you cite next, which specify the nature of that reliance.

- Again, the reviewer is spot on that there's a difference between the gravity of the pure research vs the general reach and accessibility of WEF. The intent was to use WEF a hook, but the research as an intellectual anchor. Do you think we should drop the WEF reference?

Where you write "Yet action on biodiversity policy and finance has long lagged climate efforts."
This is a critical and excellent point and so I'm glad to see this so well argued

- Thank you - this has been a major source of frustration for a very long time.

Where you write “The results in Fig 1 reveal an important feature of the nature-GDP-credit ratings197relationship, which is highly non-linear. This is demonstrated in the rankings of198countries by impacts on GDP versus credit ratings. In terms of impacts on GDP, China199and Malaysia are in the middle of the pack, with shortfalls of 3.1% and 4.2%,200respectively (Fig 1, Panel A). But these translate into the greatest impacts on201sovereign downgrades, with China and Malaysia sliding 5.5 and 6.3 notches,202” The nonlinearity is a hugely important point. I would expand and clarify this section, possibly with a plot of a “degree of nonlinearity” derived from the ratio between the scenarios.

- Another apt point. The non-linearity is a feature of the way macroeconomic fundamentals drive rating assessment, rather than anything arising from the scenarios we have modelled. Consider a rich economy, where investors do not reasonably expect any probability of default within the next ten years and has a AAA rating. What rating would the rating agencies give to an even richer economy? This simple thought experiment highlights an important feature of the data-generating process in rating assignments.

  Annually, S&P publish GDP “bandings”, real levels in GDP which correspond to certain ranges on the rating scale. These bandings create an almost step-like feature in the data, which we demonstrate in Figure M.3. The non-linearity we refer to arises directly from the methodologies use by ratings agencies and the structure of sovereign credit ratings. Credit rating agencies assess macroeconomic fundamentals using discrete rating bands or “buckets”, including for income and development indicators such as GDP per capita. These bands introduce an inherent non-linearity into the mapping between GDP and ratings: countries located near the centre of a band may experience moderate GDP changes with little effect on their rating, whereas countries positioned near a threshold between bands can experience large rating changes in response to relatively small GDP shocks.

- Our methodology molds around this data generating process and reveals these non-linearities to us. The random-forest model recovers these threshold effects directly from the historical ratings data, revealing a step-like relationship between GDP and credit ratings rather than a smooth linear gradient. This explains why countries with similar GDP impacts can experience very different rating outcomes, and why some countries (e.g. China) appear particularly sensitive to modest GDP losses.

- In response to this comment, we have made direct changes to the text. For convenience, we have pasted the offending paragraph here and underlined the new text: **P7, L1 – L13**

“The results in Fig 1 reveal an important feature of the nature-GDP-credit ratings relationship, which is highly non-linear. This is demonstrated in the rankings of countries by impacts on GDP versus credit ratings. <u>Non-linearity in the GDP–ratings relationship arises because ratings are constructed using discrete ‘income bands’. Countries near band thresholds can experience large rating changes from modest GDP shocks, whereas countries in the middle of a band show limited</u>

sensitivity. Our model recovers these threshold effects directly from the ratings data, explaining the observed cross-country heterogeneity. In terms of impacts on GDP, China and Malaysia are in the middle of the pack, with shortfalls of 3.1% and 4.2%, respectively (Fig 1, Panel A). But these translate into the greatest impacts on sovereign downgrades, with China and Malaysia sliding 5.5 and 6.3 notches, respectively (Fig 1, Panel B). Finally, because these results reflect losses across only a limited range of ecosystem services, the associated impacts should be considered lower-bounds."

- Further discussion of non-linearities is also found in the supplementary material text around figure M3 (**P15, L24 – P16, L9)**.

Regarding "For investors, we show that forward-looking environmental risks can be incorporated277into mainstream risk measures such as credit ratings, without the need to resort to278poorly defined 'ESG' metrics that often lack scientific underpinnings.279"
This is very important for many practitioners in hear the message that ESG tools are often very flawed. I would suggest emphasizing this more and earlier in the text.

- Agreed. Though admittedly ESG seems to be dying its own death these days without much input from us. The challenge is that we're already 200 words over the limit for an Article, so would be reluctant to introduce duplications. In our experience, intro and discussion are generally the most prominent sections, so hopefully others will find as you did.

  What I'd really like to put into the text is the following, but we have to respect the journal's limits:

  Sovereign credit ratings answer the following question: 'what is the ability and willingness of this sovereign to service its debt?'. Markets may think ratings agencies have better or worse answers, but that is the question. When it comes to ESG, no one has been able to articulate to me precisely what question these metrics are trying to answer. Lots of people have told me they've answered it, but none of them can tell me what the underlying question really is. We didn't want to wade into that swamp. If you think science tells us there's a macro-material financial risk whereby nature-loss affects the sovereign's ability to service its debt, then it's fair-game to expect the mainstream sovereign rating to incorporate it. We think we've found a way to make that happen (or at least partially reduce the gap).

Regarding: "This implementation entails harmonising GTAP–InVEST AEZ-region shocks with362national accounts, propagating nature-adjusted GDP into fiscal/external indicators via363practitioner-based mappings, and embedding the resulting macro paths in a ratings–364probability of default–costs of borrowing pipeline.365
This is the only part of the manuscript where I was confused. The text suggests you are using

the "shocks" that went into GTAP-InVEST as inputs to your subsequent calculations? I thought it was the GDP results FROM GTAP-InVEST that were the primary inputs. Please clarify.

- Thanks. And sorry for that rather monstrous sentence. You are correct, we use outputs (nature-adjusted GDP) from GTAP-InVEST as inputs into our model. We've deleted the redundant clause the text now reads:

We then integrate forward-looking macroeconomic paths derived from GTAP–InVEST via a sovereign-level aggregation and indicator-adjustment layer, linking scenario outputs to the six ratings determinants in our prediction model. This implementation entails propagating nature-adjusted GDP (from GTAP-InVEST) into fiscal/external indicators via practitioner-based mappings, and embedding the resulting nature-adjusted GDP and nature-adjusted fiscal indicators into a ratings–probability of default–costs of borrowing pipeline.

Reviewer #3 (Remarks on code availability):

The Github Repo is very well implemented and organized.

- Thank you, both for this supportive comment but also for making the dedicated effort to examine this in detail. We are delighted the replication package meets your high standards.

References used in responses to all referees.

1. Johnson, J. A. *et al. The Economic Case for Nature*. (World Bank, Washington, DC, 2021). doi:10.1596/35882.

2. Johnson, J. A. *et al.* Investing in nature can improve equity and economic returns. *Proc. Natl. Acad. Sci.* **120**, e2220401120 (2023).

3. Weitzman, M. L. Fat-Tailed Uncertainty in the Economics of Catastrophic Climate Change. *Rev. Environ. Econ. Policy* **5**, 275–292 (2011).

4. Ranger, N. *et al. The Green Scorpion: The Macro-Criticality of Nature for Finance – Foundations for Scenario-Based Analysis of Complex and Cascading Physical Nature-Related Risks*. (2023).

5. Dietz, S., Bowen, A., Dixon, C. & Gradwell, P. 'Climate value at risk' of global financial assets. *Nat. Clim. Change* **6**, 676–679 (2016).

6. Semieniuk, G. *et al.* Stranded fossil-fuel assets translate to major losses for investors in advanced economies. *Nat. Clim. Change* **12**, 532–538 (2022).

7. Szuwalski, C. S., Aydin, K., Fedewa, E. J., Garber-Yonts, B. & Litzow, M. A. The collapse of eastern Bering Sea snow crab. *Science* **382**, 306–310 (2023).

8. Melnikova, I. *et al.* Amazon dieback beyond the 21st century under high-emission scenarios by Earth System models. *Commun. Earth Environ.* **6**, 670 (2025).

9. Amigo, I. When will the Amazon hit a tipping point? *Nature* **578**, 505–507 (2020).

10. Flores, B. M. *et al.* Critical transitions in the Amazon forest system. *Nature* **626**, 555–564 (2024).

11. Lovejoy, T. E. & Nobre, C. Amazon tipping point: Last chance for action. *Sci. Adv.* **5**, eaba2949 (2019).

12. Lovejoy, T. E. & Nobre, C. Amazon Tipping Point. *Sci. Adv.* **4**, eaat2340 (2018).

13. Feuerbacher, A., Kempen, M., Steidle, J. L. M. & Wieck, C. The economic, agricultural, and food security repercussions of a wild pollinator collapse in Europe. *Nat. Commun.* **16**, 9892 (2025).

14. Millard, J. *et al.* Key tropical crops at risk from pollinator loss due to climate change and land use. *Sci. Adv.* **9**, eadh0756 (2023).

15. Janousek, W. M. *et al.* Recent and future declines of a historically widespread pollinator linked to climate, land cover, and pesticides. *Proc. Natl. Acad. Sci.* **120**, e2211223120 (2023).

16. Bioy, H. Global ESG Mutual Fund and ETF Funds Register Outflows in Q3 2025 Against a Complex Geopolitical Backdrop. *Morningstar, Inc.* https://www.morningstar.com/sustainable-investing/global-esg-mutual-fund-etf-funds-register-outflows-q3-2025-against-complex-geopolitical-backdrop (2025).

17. Agarwala, M. & Coyle, D. Natural capital in climate models. *Nat. Sustain.* **4**, 81–82 (2021).

18. Bastien-Olvera, B. A. *et al.* Unequal climate impacts on global values of natural capital. *Nature* **625**, 722–727 (2024).

19. Bastien-Olvera, B. A. & Moore, F. C. Use and non-use value of nature and the social cost of carbon. *Nat. Sustain.* **4**, 101–108 (2021).

20. Bastien-Olvera, B. A. & Moore, F. C. Climate Impacts on Natural Capital: Consequences for the Social Cost of Carbon. *Annu. Rev. Resour. Econ.* **14**, 515–532 (2022).

21. Tran, Y., Vu, H., Klusak, P., Kraemer, M. & Hoang, T. Sovereign credit ratings during the COVID-19 pandemic. *Int. Rev. Financ. Anal.* **78**, 101879 (2021).
